# Supplementary material for: TCGA Expression Analyses of 10 Carcinoma Types Reveal Clinically Significant Racial Differences
Source: Cancers (Basel). 2023 May 10;15(10):2695. doi: 10.3390/cancers15102695 (PMC10216530; doi:10.3390/cancers15102695)
Supplement: Supplementary file 1 [file cancers-15-02695-s001.zip › cancers-2270182-Supplemental S5.pdf]

Reactome Analysis Result

14 January, 2021

Introduction

This report summarizes the pathway analysis result created by the **Reactome Analysis Service**. For more information, visit https://www.reactome.org.

In this reports, pathways found differentially expressed at an adjusted p-value <= 0.05 are considered to be significantly regulated.

Analysis Overview

- Number of datasets analysed: 9
- Reactome version: 74
- Disease pathways were included

Dataset summaries

CORE:

- 987 pathways
- 130 fold changes for genes / proteins

UCEC:

- 987 pathways
- 130 fold changes for genes / proteins

BRCA:

- 987 pathways
- 130 fold changes for genes / proteins

KIRC:

- 987 pathways
- 130 fold changes for genes / proteins

KIRP:

- 987 pathways
- 130 fold changes for genes / proteins

LUAD:

- 987 pathways
- 130 fold changes for genes / proteins

LIHC:

- 987 pathways
- 130 fold changes for genes / proteins

STAD:

- 987 pathways
- 130 fold changes for genes / proteins

THCA:

- 987 pathways
- 130 fold changes for genes / proteins

Number of datasets the different pathways were found to be significantly regulated in:

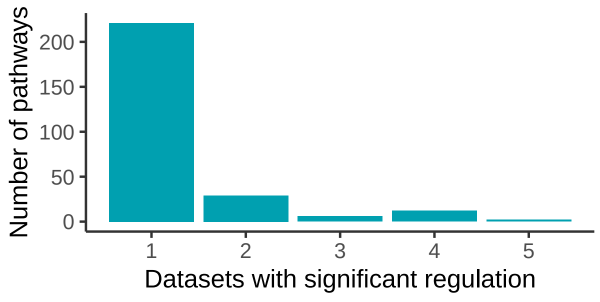

Pathway analysis

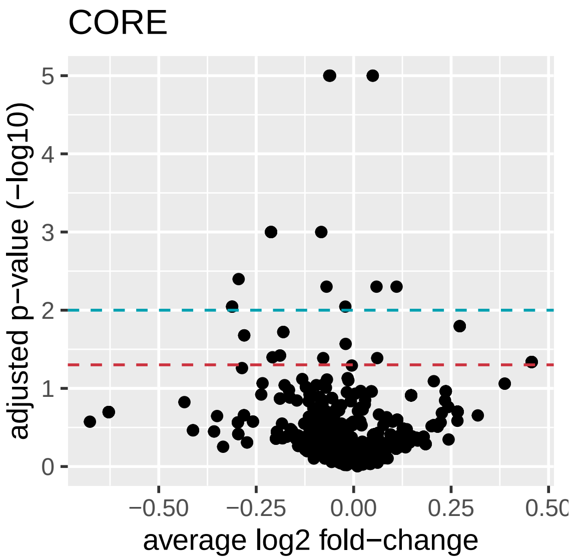

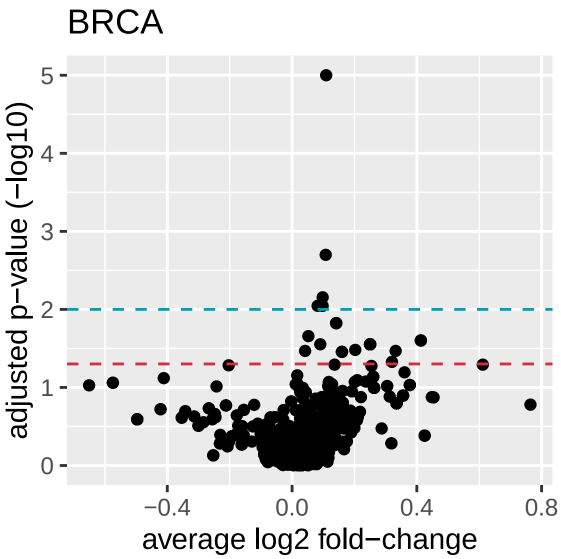

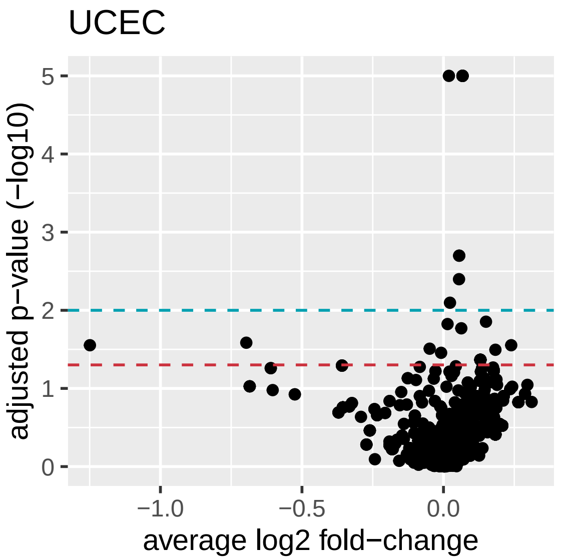

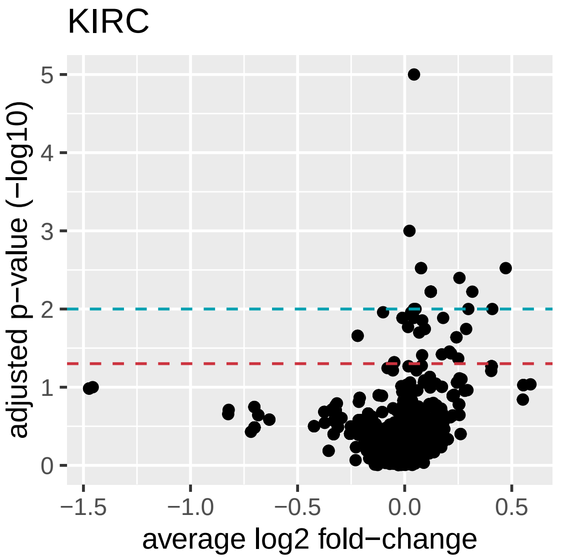

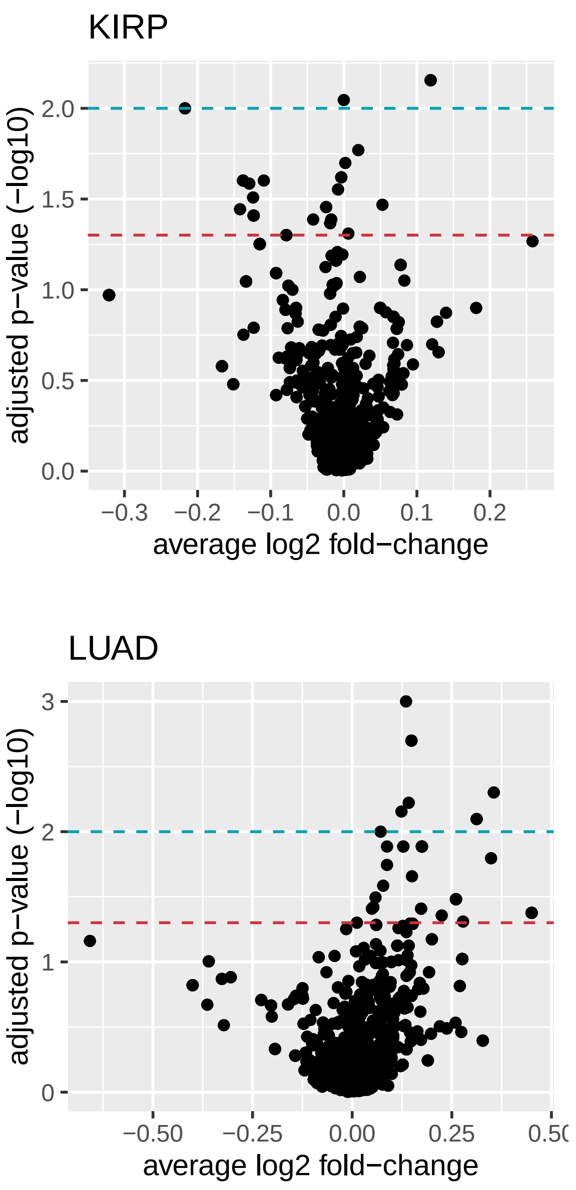
KIRP
LIHC
LUAD
STAD
adjusted p−value (−log10)
average log2 fold−change

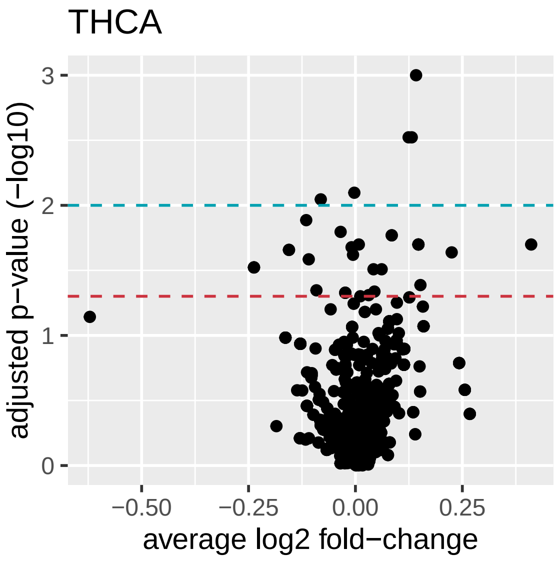

These volcano plots summarize the pathway results for every dataset. Every point is one pathway. The x-axis represents the average fold-change of all genes / proteins within that pathway. The y-axis represents the p-value where "higher" values are more significant (-log10 transformation). The red line represents p = 0.05, and the blue line p = 0.01.

Pathway correlation

Correlation between all datasets (if more than one). Every point represents one pathway, with the x-axis showing the average fold-change of one dataset and the y-axis the average fold-change of the other dataset. Only pathways that were observed in both datasets are shown. Points are colored based on the lowest observed significance.

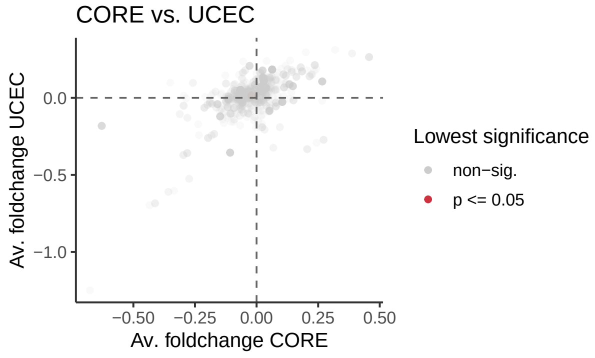

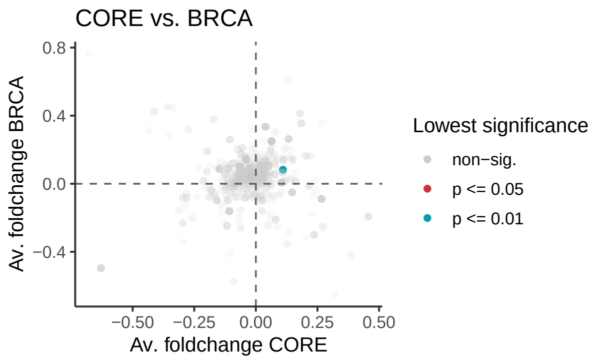

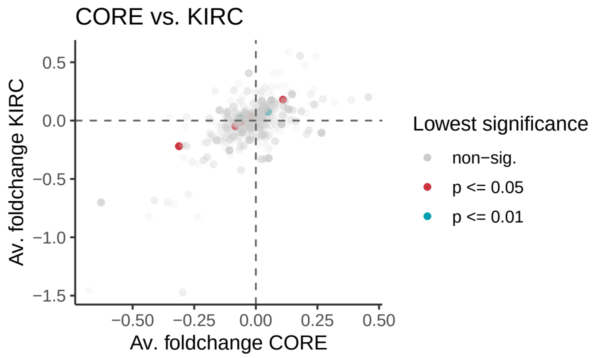

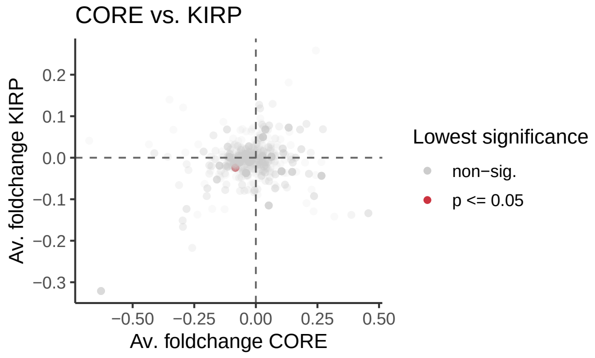

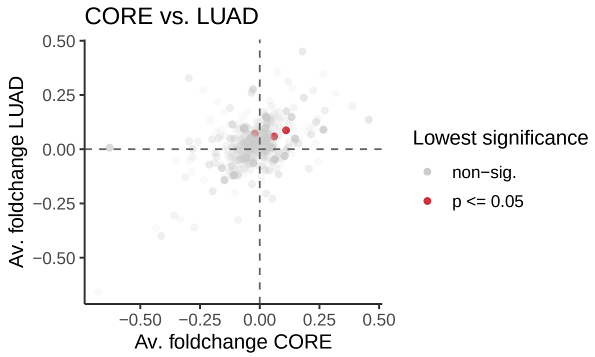

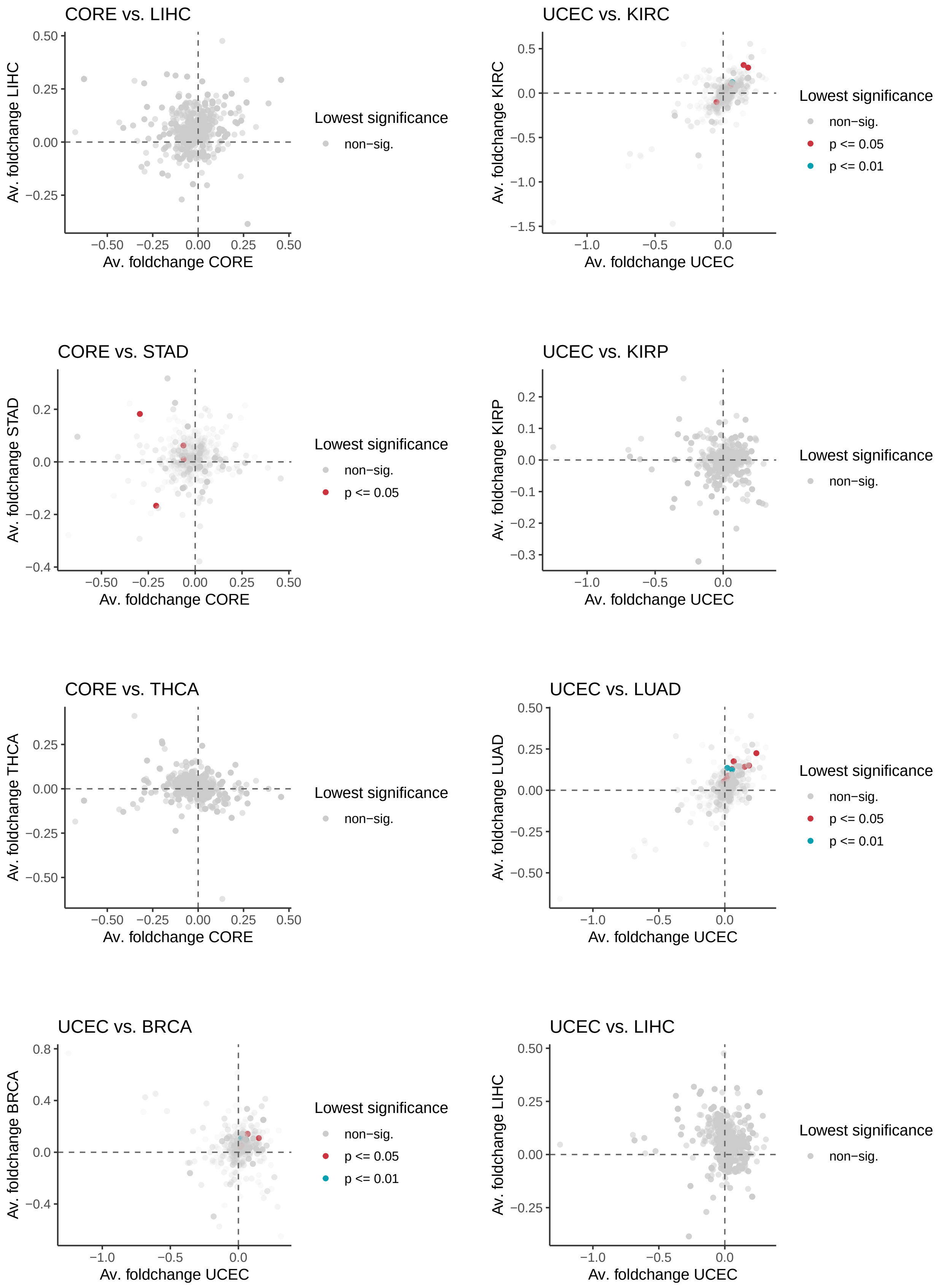
CORE vs. LIHC
Av. foldchange LIHC
Av. foldchange CORE
Lowest significance
non−sig.
UCEC vs. KIRC
Av. foldchange KIRC
Av. foldchange UCEC
Lowest significance
non−sig.
p <= 0.05
p <= 0.01
CORE vs. STAD
Av. foldchange STAD
Av. foldchange CORE
Lowest significance
non−sig.
p <= 0.05
UCEC vs. KIRP
Av. foldchange KIRP
Av. foldchange UCEC
Lowest significance
non−sig.
CORE vs. THCA
Av. foldchange THCA
Av. foldchange CORE
Lowest significance
non−sig.
UCEC vs. LUAD
Av. foldchange LUAD
Av. foldchange UCEC
Lowest significance
non−sig.
p <= 0.05
p <= 0.01
UCEC vs. BRCA
Av. foldchange BRCA
Av. foldchange UCEC
Lowest significance
non−sig.
p <= 0.05
p <= 0.01
UCEC vs. LIHC
Av. foldchange LIHC
Av. foldchange UCEC
Lowest significance
non−sig.

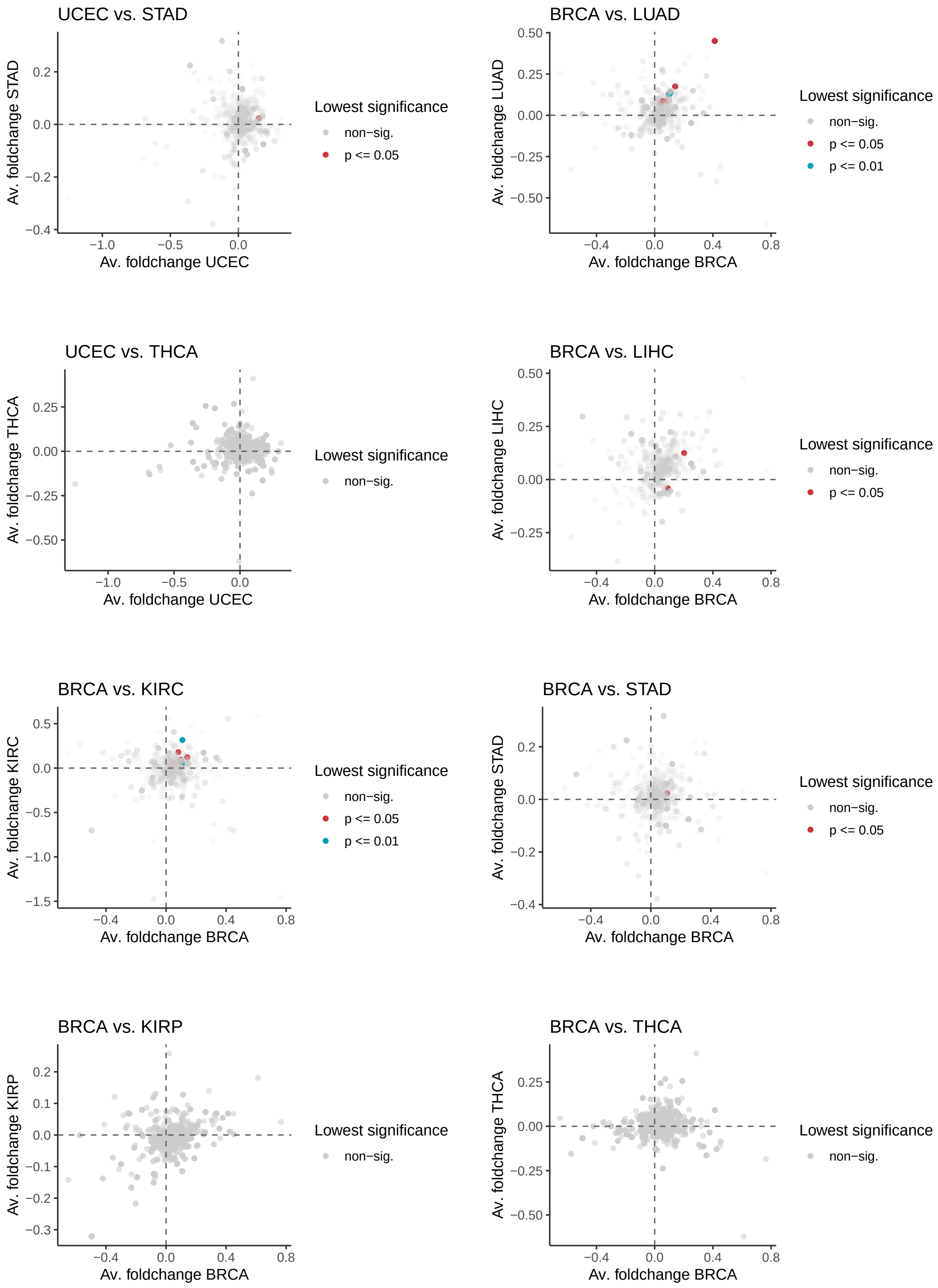
UCEC vs. STAD
Av. foldchange STAD
Av. foldchange UCEC
Lowest significance
non−sig.
p <= 0.05
BRCA vs. LUAD
Av. foldchange LUAD
Av. foldchange BRCA
Lowest significance
non−sig.
p <= 0.05
p <= 0.01
UCEC vs. THCA
Av. foldchange THCA
Av. foldchange UCEC
Lowest significance
non−sig.
BRCA vs. LIHC
Av. foldchange LIHC
Av. foldchange BRCA
Lowest significance
non−sig.
p <= 0.05
BRCA vs. KIRC
Av. foldchange KIRC
Av. foldchange BRCA
Lowest significance
non−sig.
p <= 0.05
p <= 0.01
BRCA vs. STAD
Av. foldchange STAD
Av. foldchange BRCA
Lowest significance
non−sig.
p <= 0.05
BRCA vs. KIRP
Av. foldchange KIRP
Av. foldchange BRCA
Lowest significance
non−sig.
BRCA vs. THCA
Av. foldchange THCA
Av. foldchange BRCA
Lowest significance
non−sig.

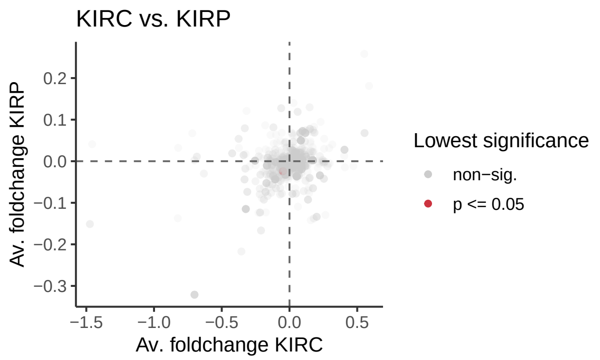
KIRC vs. KIRP
Av. foldchange KIRP
Av. foldchange KIRC
Lowest significance
non−sig.
p <= 0.05

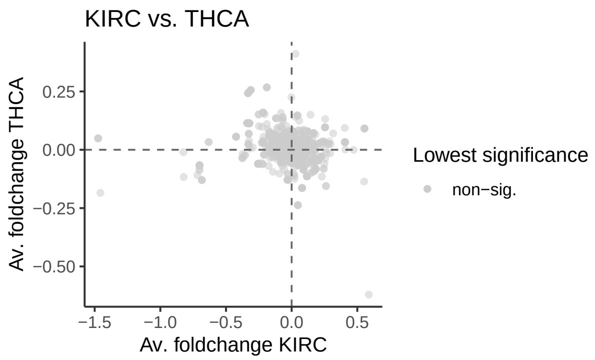
KIRC vs. THCA
Av. foldchange THCA
Av. foldchange KIRC
Lowest significance
non−sig.

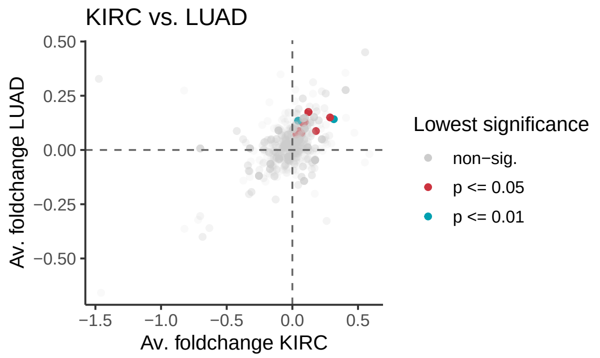
KIRC vs. LUAD
Av. foldchange LUAD
Av. foldchange KIRC
Lowest significance
non−sig.
p <= 0.05
p <= 0.01

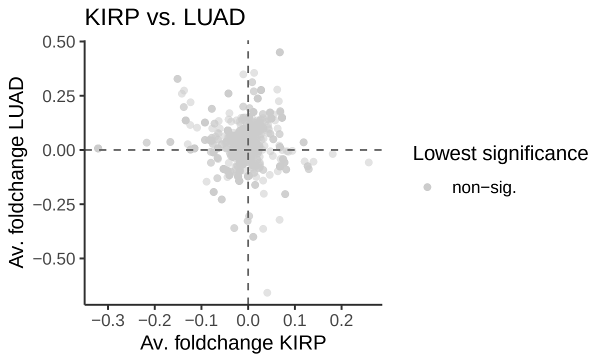
KIRP vs. LUAD
Av. foldchange LUAD
Av. foldchange KIRP
Lowest significance
non−sig.

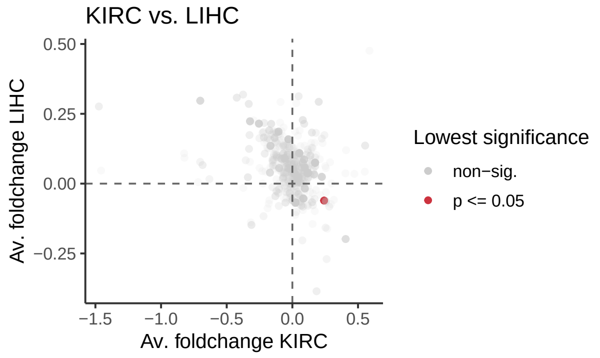
KIRC vs. LIHC
Av. foldchange LIHC
Av. foldchange KIRC
Lowest significance
non−sig.
p <= 0.05

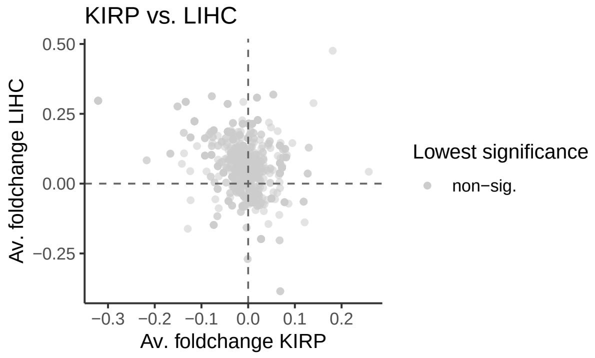
KIRP vs. LIHC
Av. foldchange LIHC
Av. foldchange KIRP
Lowest significance
non−sig.

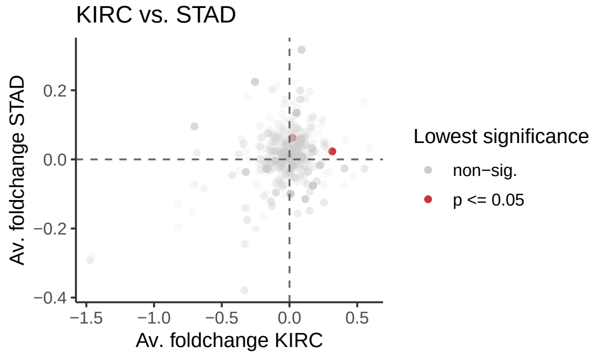
KIRC vs. STAD
Av. foldchange STAD
Av. foldchange KIRC
Lowest significance
non−sig.
p <= 0.05

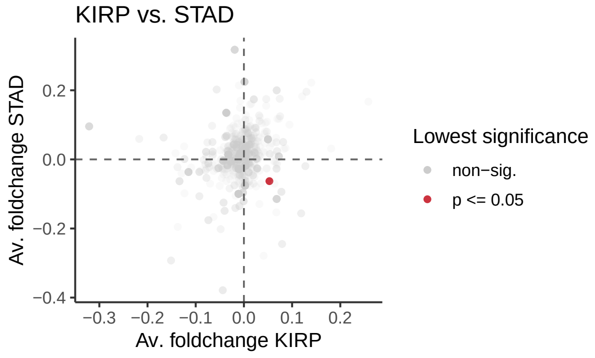
KIRP vs. STAD
Av. foldchange STAD
Av. foldchange KIRP
Lowest significance
non−sig.
p <= 0.05

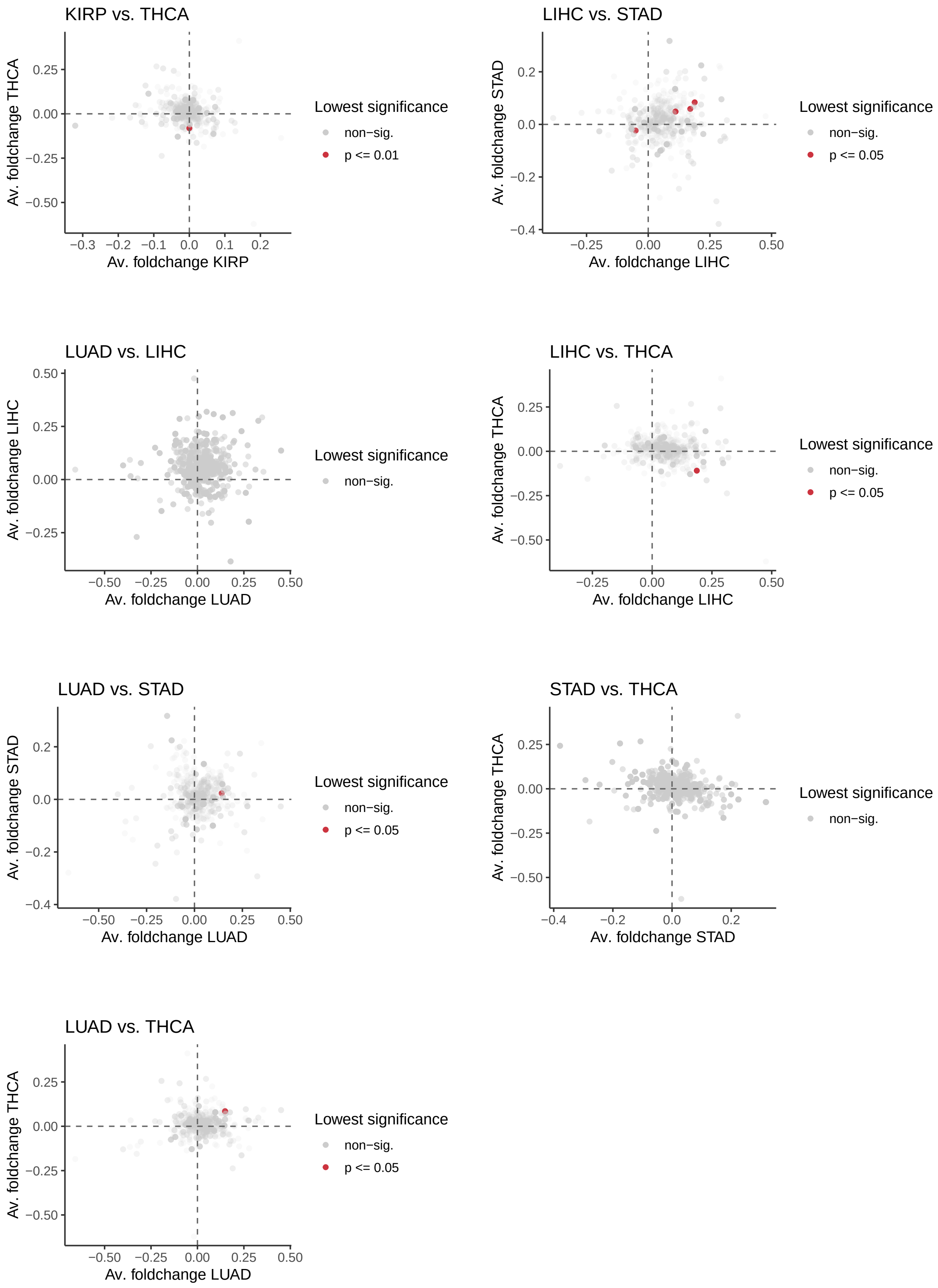

KIRP vs. THCA
Av. foldchange THCA
0.25
0.00
−0.25
−0.50
−0.3 −0.2 −0.1 0.0 0.1 0.2
Av. foldchange KIRP
Lowest significance
non−sig.
p <= 0.01
LIHC vs. STAD
Av. foldchange STAD
0.2
0.0
−0.2
−0.4
−0.25 0.00 0.25 0.50
Av. foldchange LIHC
Lowest significance
non−sig.
p <= 0.05
LUAD vs. LIHC
Av. foldchange LIHC
0.50
0.25
0.00
−0.25
−0.50 −0.25 0.00 0.25 0.50
Av. foldchange LUAD
Lowest significance
non−sig.
LIHC vs. THCA
Av. foldchange THCA
0.25
0.00
−0.25
−0.50
−0.25 0.00 0.25 0.50
Av. foldchange LIHC
Lowest significance
non−sig.
p <= 0.05
LUAD vs. STAD
Av. foldchange STAD
0.2
0.0
−0.2
−0.4
−0.50 −0.25 0.00 0.25 0.50
Av. foldchange LUAD
Lowest significance
non−sig.
p <= 0.05
STAD vs. THCA
Av. foldchange THCA
0.25
0.00
−0.25
−0.50
−0.4 −0.2 0.0 0.2
Av. foldchange STAD
Lowest significance
non−sig.
LUAD vs. THCA
Av. foldchange THCA
0.25
0.00
−0.25
−0.50
−0.50 −0.25 0.00 0.25 0.50
Av. foldchange LUAD
Lowest significance
non−sig.
p <= 0.05

Similar regulated pathways

Pathways that show the same direction on all of the datasets. The direction of regulation is only assessed for significantly regulated pathways.

Nuclear Receptor transcription pathway (R-HSA-383280)

	CORE	UCEC	BRCA	KIRC	KIRP	LUAD	LIHC	STAD	THCA
Regulation	**Up**	Down	Down	**Up**	Up	Up	Down	Up	Down
FDR	**< 0.001**	0.945	0.24	**0.003**	0.35	0.196	0.206	0.051	0.918
Av. FC	0.049	-0.088	-0.246	0.076	0.067	0.073	-0.203	0.049	-0.029
N Genes	3	3	3	3	3	3	3	3	3

SUMOylation of intracellular receptors (R-HSA-4090294)

	CORE	UCEC	BRCA	KIRC	KIRP	LUAD	LIHC	STAD	THCA
Regulation	**Up**	Down	Down	**Up**	Up	Up	Down	Up	Down
FDR	**< 0.001**	0.945	0.24	**0.003**	0.35	0.196	0.206	0.051	0.918
Av. FC	0.049	-0.088	-0.246	0.076	0.067	0.073	-0.203	0.049	-0.029
N Genes	3	3	3	3	3	3	3	3	3

Estrogen-dependent gene expression (R-HSA-9018519)

	CORE	UCEC	BRCA	KIRC	KIRP	LUAD	LIHC	STAD	THCA
Regulation	**Up**	Down	Down	Up	Up	Up	Down	Down	Down
FDR	**0.005**	0.897	0.396	0.053	0.898	0.463	0.446	0.532	0.485
Av. FC	0.059	-0.048	-0.178	0.08	0.016	0.07	-0.095	-0.007	-0.031
N Genes	6	6	6	6	6	6	6	6	6

Nuclear signaling by ERBB4 (R-HSA-1251985)

	CORE	UCEC	BRCA	KIRC	KIRP	LUAD	LIHC	STAD	THCA
Regulation	**Up**	Up	**Up**	**Up**	Up	**Up**	Down	Up	Down
FDR	**0.005**	0.672	**0.009**	**0.013**	0.481	**0.018**	0.609	0.567	0.104
Av. FC	0.11	0.011	0.082	0.18	0.023	0.087	-0.023	0.048	-0.006
N Genes	6	6	6	6	6	6	6	6	6

RUNX1 regulates estrogen receptor mediated transcription (R-HSA-8931987)

	CORE	UCEC	BRCA	KIRC	KIRP	LUAD	LIHC	STAD	THCA
Regulation	**Up**	Down	Down	Up	Up	Up	Down	Up	Down
FDR	**0.016**	0.523	0.741	0.343	0.311	0.16	0.195	0.654	0.446
Av. FC	0.272	-0.272	-0.253	0.184	0.069	0.178	-0.385	0.024	-0.083
N Genes	1	1	1	1	1	1	1	1	1

RUNX1 regulates transcription of genes involved in WNT signaling (R-HSA-8939256)

	CORE	UCEC	BRCA	KIRC	KIRP	LUAD	LIHC	STAD	THCA
Regulation	**Up**	Down	Down	Up	Up	Up	Down	Up	Down
FDR	**0.016**	0.523	0.741	0.343	0.311	0.16	0.195	0.654	0.446
Av. FC	0.272	-0.272	-0.253	0.184	0.069	0.178	-0.385	0.024	-0.083
N Genes	1	1	1	1	1	1	1	1	1

Regulation of RUNX2 expression and activity (R-HSA-8939902)

	CORE	UCEC	BRCA	KIRC	KIRP	LUAD	LIHC	STAD	THCA
Regulation	**Up**	Down	Down	Up	Up	Up	Down	Up	Down
FDR	**0.016**	0.523	0.741	0.343	0.311	0.16	0.195	0.654	0.446
Av. FC	0.272	-0.272	-0.253	0.184	0.069	0.178	-0.385	0.024	-0.083
N Genes	1	1	1	1	1	1	1	1	1

Signaling by ERBB4 (R-HSA-1236394)

	CORE	UCEC	BRCA	KIRC	KIRP	LUAD	LIHC	STAD	THCA
Regulation	**Up**	Up	Up	Up	Up	**Up**	Down	Up	Up
FDR	**0.041**	0.464	0.181	0.101	0.704	**0.032**	0.882	0.865	0.281
Av. FC	0.06	0.049	0.056	0.12	0.026	0.059	-0.013	0.032	0.013
N Genes	10	10	10	10	10	10	10	10	10

Biotin transport and metabolism (R-HSA-196780)

	CORE	UCEC	BRCA	KIRC	KIRP	LUAD	LIHC	STAD	THCA
Regulation	**Up**	Up	Down	Up	Down	Up	Up	Down	Down
FDR	**0.046**	0.15	0.421	0.463	0.09	0.469	0.285	0.641	0.587
Av. FC	0.457	0.265	-0.193	0.201	-0.134	0.136	0.293	-0.063	-0.046
N Genes	1	1	1	1	1	1	1	1	1

Carnitine metabolism (R-HSA-200425)

	CORE	UCEC	BRCA	KIRC	KIRP	LUAD	LIHC	STAD	THCA
Regulation	**Up**	Up	Down	Up	Down	Up	Up	Down	Down
FDR	**0.046**	0.15	0.421	0.463	0.09	0.469	0.285	0.641	0.587
Av. FC	0.457	0.265	-0.193	0.201	-0.134	0.136	0.293	-0.063	-0.046
N Genes	1	1	1	1	1	1	1	1	1

Defects in vitamin and cofactor metabolism (R-HSA-3296482)

	CORE	UCEC	BRCA	KIRC	KIRP	LUAD	LIHC	STAD	THCA
Regulation	**Up**	Up	Down	Up	Down	Up	Up	Down	Down
FDR	**0.046**	0.15	0.421	0.463	0.09	0.469	0.285	0.641	0.587
Av. FC	0.457	0.265	-0.193	0.201	-0.134	0.136	0.293	-0.063	-0.046
N Genes	1	1	1	1	1	1	1	1	1

Defects in biotin (Btn) metabolism (R-HSA-3323169)

	CORE	UCEC	BRCA	KIRC	KIRP	LUAD	LIHC	STAD	THCA
Regulation	**Up**	Up	Down	Up	Down	Up	Up	Down	Down
FDR	**0.046**	0.15	0.421	0.463	0.09	0.469	0.285	0.641	0.587
Av. FC	0.457	0.265	-0.193	0.201	-0.134	0.136	0.293	-0.063	-0.046
N Genes	1	1	1	1	1	1	1	1	1

Defective HLCS causes multiple carboxylase deficiency (R-HSA-3371599)

	CORE	UCEC	BRCA	KIRC	KIRP	LUAD	LIHC	STAD	THCA
Regulation	**Up**	Up	Down	Up	Down	Up	Up	Down	Down
FDR	**0.046**	0.15	0.421	0.463	0.09	0.469	0.285	0.641	0.587
Av. FC	0.457	0.265	-0.193	0.201	-0.134	0.136	0.293	-0.063	-0.046
N Genes	1	1	1	1	1	1	1	1	1

Vasopressin regulates renal water homeostasis via Aquaporins (R-HSA-432040)

	CORE	UCEC	BRCA	KIRC	KIRP	LUAD	LIHC	STAD	THCA
Regulation	**Down**	Down	Up	Down	Up	Down	Up	Up	Up
FDR	**0.001**	0.366	0.944	0.193	0.592	0.36	0.972	0.382	0.127
Av. FC	-0.212	-0.064	0.014	-0.339	0.015	-0.071	0.023	0.044	0.114
N Genes	1	1	1	1	1	1	1	1	1

Aquaporin-mediated transport (R-HSA-445717)

	CORE	UCEC	BRCA	KIRC	KIRP	LUAD	LIHC	STAD	THCA
Regulation	**Down**	Down	Up	Down	Up	Down	Up	Up	Up
FDR	**0.001**	0.366	0.944	0.193	0.592	0.36	0.972	0.382	0.127
Av. FC	-0.212	-0.064	0.014	-0.339	0.015	-0.071	0.023	0.044	0.114
N Genes	1	1	1	1	1	1	1	1	1

VxPx cargo-targeting to cilium (R-HSA-5620916)

	CORE	UCEC	BRCA	KIRC	KIRP	LUAD	LIHC	STAD	THCA
Regulation	**Down**	Down	Up	Down	Up	Down	Up	Up	Up
FDR	**0.001**	0.366	0.944	0.193	0.592	0.36	0.972	0.382	0.127
Av. FC	-0.212	-0.064	0.014	-0.339	0.015	-0.071	0.023	0.044	0.114
N Genes	1	1	1	1	1	1	1	1	1

Cargo trafficking to the periciliary membrane (R-HSA-5620920)

	CORE	UCEC	BRCA	KIRC	KIRP	LUAD	LIHC	STAD	THCA
Regulation	**Down**	Down	Up	Down	Up	Down	Up	Up	Up
FDR	**0.001**	0.366	0.944	0.193	0.592	0.36	0.972	0.382	0.127
Av. FC	-0.212	-0.064	0.014	-0.339	0.015	-0.071	0.023	0.044	0.114
N Genes	1	1	1	1	1	1	1	1	1

Cellular responses to stress (R-HSA-2262752)

	CORE	UCEC	BRCA	KIRC	KIRP	LUAD	LIHC	STAD	THCA
Regulation	**Down**	Up	Down	**Down**	**Down**	Up	Up	Up	Up
FDR	**0.001**	0.524	0.151	**0.048**	**0.035**	0.102	0.066	0.149	0.694
Av. FC	-0.083	0.003	-0.003	-0.049	-0.024	0.068	0.059	0.027	0.047
N Genes	21	21	21	21	21	21	21	21	21

Cellular responses to external stimuli (R-HSA-8953897)

	CORE	UCEC	BRCA	KIRC	KIRP	LUAD	LIHC	STAD	THCA
Regulation	**Down**	Up	Down	**Down**	**Down**	Up	Up	Up	Up
FDR	**0.001**	0.524	0.151	**0.048**	**0.035**	0.102	0.066	0.149	0.694
Av. FC	-0.083	0.003	-0.003	-0.049	-0.024	0.068	0.059	0.027	0.047
N Genes	21	21	21	21	21	21	21	21	21

Post-translational protein modification (R-HSA-597592)

	CORE	UCEC	BRCA	KIRC	KIRP	LUAD	LIHC	STAD	THCA
Regulation	**Down**	Down	Down	Down	Up	Up	Up	Up	Up
FDR	**0.005**	0.872	0.361	0.167	0.704	0.836	0.669	0.085	0.517
Av. FC	-0.07	-0.028	-0.08	-0.008	0.001	0.028	0	0.009	0.035
N Genes	22	22	22	22	22	22	22	22	22

Formation of Incision Complex in GG-NER (R-HSA-5696395)

	CORE	UCEC	BRCA	KIRC	KIRP	LUAD	LIHC	STAD	THCA
Regulation	**Down**	Down	Down	**Down**	Down	Down	Down	Up	Down
FDR	**0.009**	0.274	0.194	**0.022**	0.135	0.176	0.689	0.527	0.427
Av. FC	-0.312	-0.105	-0.155	-0.22	-0.066	-0.13	-0.117	0.097	-0.061
N Genes	1	1	1	1	1	1	1	1	1

Fanconi Anemia Pathway (R-HSA-6783310)

	CORE	UCEC	BRCA	KIRC	KIRP	LUAD	LIHC	STAD	THCA
Regulation	**Down**	Down	Down	**Down**	Down	Down	Down	Up	Down
FDR	**0.009**	0.274	0.194	**0.022**	0.135	0.176	0.689	0.527	0.427
Av. FC	-0.312	-0.105	-0.155	-0.22	-0.066	-0.13	-0.117	0.097	-0.061
N Genes	1	1	1	1	1	1	1	1	1

Metabolism of proteins (R-HSA-392499)

	CORE	UCEC	BRCA	KIRC	KIRP	LUAD	LIHC	STAD	THCA
Regulation	**Down**	Up	Down	Up	Down	Up	Up	Up	Up
FDR	**0.009**	0.455	0.586	0.182	0.444	0.776	0.912	0.106	0.68
Av. FC	-0.021	0.008	-0.046	0.02	-0.007	0.041	0.012	0.005	0.024
N Genes	32	32	32	32	32	32	32	32	32

Nucleotide Excision Repair (R-HSA-5696398)

	CORE	UCEC	BRCA	KIRC	KIRP	LUAD	LIHC	STAD	THCA
Regulation	**Down**	Down	Down	Down	Down	Down	Down	Down	Up
FDR	**0.019**	0.494	0.336	0.127	0.44	0.157	0.926	0.562	0.325
Av. FC	-0.18	-0.039	-0.036	-0.121	-0.02	-0.036	-0.027	-0.009	0.014
N Genes	3	3	3	3	3	3	3	3	3

Global Genome Nucleotide Excision Repair (GG-NER) (R-HSA-5696399)

	CORE	UCEC	BRCA	KIRC	KIRP	LUAD	LIHC	STAD	THCA
Regulation	**Down**	Down	Down	Down	Down	Down	Down	Down	Up
FDR	**0.019**	0.494	0.336	0.127	0.44	0.157	0.926	0.562	0.325
Av. FC	-0.18	-0.039	-0.036	-0.121	-0.02	-0.036	-0.027	-0.009	0.014
N Genes	3	3	3	3	3	3	3	3	3

Transcription-Coupled Nucleotide Excision Repair (TC-NER) (R-HSA-6781827)

	CORE	UCEC	BRCA	KIRC	KIRP	LUAD	LIHC	STAD	THCA
Regulation	**Down**	Down	Down	Down	Down	Down	Down	Down	Up
FDR	**0.019**	0.494	0.336	0.127	0.44	0.157	0.926	0.562	0.325
Av. FC	-0.18	-0.039	-0.036	-0.121	-0.02	-0.036	-0.027	-0.009	0.014
N Genes	3	3	3	3	3	3	3	3	3

TFAP2 (AP-2) family regulates transcription of cell cycle factors (R-HSA-8866911)

	CORE	UCEC	BRCA	KIRC	KIRP	LUAD	LIHC	STAD	THCA
Regulation	**Down**	Down	Down	Up	Down	Down	Down	Up	Up
FDR	**0.021**	0.161	0.24	0.727	0.786	0.298	0.418	0.378	0.56
Av. FC	-0.281	-0.13	-0.056	0.03	-0.016	-0.036	-0.101	0.035	0.042
N Genes	2	2	2	2	2	2	2	2	2

Transcriptional regulation of granulopoiesis (R-HSA-9616222)

	CORE	UCEC	BRCA	KIRC	KIRP	LUAD	LIHC	STAD	THCA
Regulation	**Down**	Down	Down	Up	Down	Down	Down	Up	Up
FDR	**0.021**	0.161	0.24	0.727	0.786	0.298	0.418	0.378	0.56
Av. FC	-0.281	-0.13	-0.056	0.03	-0.016	-0.036	-0.101	0.035	0.042
N Genes	2	2	2	2	2	2	2	2	2

HDR through Single Strand Annealing (SSA) (R-HSA-5685938)

	CORE	UCEC	BRCA	KIRC	KIRP	LUAD	LIHC	STAD	THCA
Regulation	Up	**Up**	**Up**	**Up**	Down	**Up**	Up	Up	Up
FDR	0.195	**< 0.001**	**0.002**	**< 0.001**	0.198	**0.001**	0.485	0.302	0.141
Av. FC	0.011	0.019	0.108	0.044	-0.007	0.135	0.025	0.059	0.063
N Genes	5	5	5	5	5	5	5	5	5

Reproduction (R-HSA-1474165)

	CORE	UCEC	BRCA	KIRC	KIRP	LUAD	LIHC	STAD	THCA
Regulation	Up	**Up**	**Up**	**Up**	Up	**Up**	Up	Up	Up
FDR	0.252	**< 0.001**	**0.015**	**0.006**	0.332	**0.013**	0.408	0.388	0.236
Av. FC	0.112	0.067	0.141	0.122	0.011	0.175	0.038	0.042	0.079
N Genes	5	5	5	5	5	5	5	5	5

Meiosis (R-HSA-1500620)

	CORE	UCEC	BRCA	KIRC	KIRP	LUAD	LIHC	STAD	THCA
Regulation	Up	**Up**	**Up**	**Up**	Up	**Up**	Up	Up	Up
FDR	0.252	**< 0.001**	**0.015**	**0.006**	0.332	**0.013**	0.408	0.388	0.236
Av. FC	0.112	0.067	0.141	0.122	0.011	0.175	0.038	0.042	0.079
N Genes	5	5	5	5	5	5	5	5	5

Resolution of D-Loop Structures (R-HSA-5693537)

	CORE	UCEC	BRCA	KIRC	KIRP	LUAD	LIHC	STAD	THCA
Regulation	Up	**Up**	**Up**	**Up**	Up	**Up**	Up	Up	Up
FDR	0.252	**< 0.001**	**0.015**	**0.006**	0.332	**0.013**	0.408	0.388	0.236
Av. FC	0.112	0.067	0.141	0.122	0.011	0.175	0.038	0.042	0.079
N Genes	5	5	5	5	5	5	5	5	5

Resolution of D-loop Structures through Synthesis-Dependent Strand Annealing (SDSA) (R-HSA-5693554)

	CORE	UCEC	BRCA	KIRC	KIRP	LUAD	LIHC	STAD	THCA
Regulation	Up	**Up**	**Up**	**Up**	Up	**Up**	Up	Up	Up
FDR	0.252	**< 0.001**	**0.015**	**0.006**	0.332	**0.013**	0.408	0.388	0.236
Av. FC	0.112	0.067	0.141	0.122	0.011	0.175	0.038	0.042	0.079
N Genes	5	5	5	5	5	5	5	5	5

Resolution of D-loop Structures through Holliday Junction Intermediates (R-HSA-5693568)

	CORE	UCEC	BRCA	KIRC	KIRP	LUAD	LIHC	STAD	THCA
Regulation	Up	**Up**	**Up**	**Up**	Up	**Up**	Up	Up	Up
FDR	0.252	**< 0.001**	**0.015**	**0.006**	0.332	**0.013**	0.408	0.388	0.236
Av. FC	0.112	0.067	0.141	0.122	0.011	0.175	0.038	0.042	0.079
N Genes	5	5	5	5	5	5	5	5	5

Meiotic recombination (R-HSA-912446)

	CORE	UCEC	BRCA	KIRC	KIRP	LUAD	LIHC	STAD	THCA
Regulation	Up	**Up**	**Up**	**Up**	Up	**Up**	Up	Up	Up
FDR	0.252	**< 0.001**	**0.015**	**0.006**	0.332	**0.013**	0.408	0.388	0.236
Av. FC	0.112	0.067	0.141	0.122	0.011	0.175	0.038	0.042	0.079
N Genes	5	5	5	5	5	5	5	5	5

Homologous DNA Pairing and Strand Exchange (R-HSA-5693579)

	CORE	UCEC	BRCA	KIRC	KIRP	LUAD	LIHC	STAD	THCA
Regulation	Up	**Up**	**Up**	**Up**	Up	**Up**	Up	Up	Up
FDR	0.109	**0.002**	**0.009**	**0.018**	0.405	**0.013**	0.468	0.125	0.149
Av. FC	0.045	0.055	0.098	0.094	0.012	0.128	0.023	0.062	0.075
N Genes	6	6	6	6	6	6	6	6	6

Presynaptic phase of homologous DNA pairing and strand exchange (R-HSA-5693616)

	CORE	UCEC	BRCA	KIRC	KIRP	LUAD	LIHC	STAD	THCA
Regulation	Up	**Up**	**Up**	**Up**	Up	**Up**	Up	Up	Up
FDR	0.109	**0.002**	**0.009**	**0.018**	0.405	**0.013**	0.468	0.125	0.149
Av. FC	0.045	0.055	0.098	0.094	0.012	0.128	0.023	0.062	0.075
N Genes	6	6	6	6	6	6	6	6	6

HDR through Homologous Recombination (HRR) (R-HSA-5685942)

	CORE	UCEC	BRCA	KIRC	KIRP	LUAD	LIHC	STAD	THCA
Regulation	Up	**Up**	**Up**	**Up**	Up	**Up**	Up	Up	Up
FDR	0.141	**0.004**	**0.007**	**0.039**	0.494	**0.007**	0.482	0.097	0.089
Av. FC	0.029	0.055	0.098	0.081	0.008	0.124	0.027	0.039	0.075
N Genes	7	7	7	7	7	7	7	7	7

HDR through Homologous Recombination (HRR) or Single Strand Annealing (SSA) (R-HSA-5693567)

	CORE	UCEC	BRCA	KIRC	KIRP	LUAD	LIHC	STAD	THCA
Regulation	Down	**Up**	**Up**	**Up**	Up	**Up**	Up	Up	Up
FDR	0.051	**0.008**	**0.022**	**0.01**	0.328	**0.013**	0.527	0.131	0.127
Av. FC	-0.005	0.023	0.052	0.051	0.008	0.087	0.023	0.052	0.04
N Genes	9	9	9	9	9	9	9	9	9

Transcriptional Regulation by E2F6 (R-HSA-8953750)

	CORE	UCEC	BRCA	KIRC	KIRP	LUAD	LIHC	STAD	THCA
Regulation	Down	**Up**	**Up**	**Up**	Up	**Up**	Down	**Up**	Up
FDR	0.098	**0.014**	**$<$ 0.001**	**0.006**	0.33	**0.006**	0.566	**0.044**	0.11
Av. FC	-0.071	0.15	0.109	0.316	0.04	0.142	-0.058	0.023	0.07
N Genes	2	2	2	2	2	2	2	2	2

Translation (R-HSA-72766)

	CORE	UCEC	BRCA	KIRC	KIRP	LUAD	LIHC	STAD	THCA
Regulation	Up	**Up**	Up	Down	Down	Up	Up	Down	Down
FDR	0.402	**0.017**	0.857	0.747	0.168	0.97	0.88	0.395	0.308
Av. FC	0.058	0.063	0.006	-0.041	-0.028	0.023	0.073	-0.024	-0.014
N Genes	5	5	5	5	5	5	5	5	5

NRAGE signals death through JNK (R-HSA-193648)

	CORE	UCEC	BRCA	KIRC	KIRP	LUAD	LIHC	STAD	THCA
Regulation	Up	**Up**	Down	Up	Up	**Up**	Up	Down	Down
FDR	0.494	**0.028**	0.481	0.928	0.3	**0.044**	0.953	0.958	0.192
Av. FC	0.041	0.239	-0.099	0.019	0.066	0.225	0.029	-0.007	-0.113
N Genes	2	2	2	2	2	2	2	2	2

PI3K/AKT activation (R-HSA-198203)

	CORE	UCEC	BRCA	KIRC	KIRP	LUAD	LIHC	STAD	THCA
Regulation	Up	**Up**	Down	**Up**	Down	**Up**	Up	Up	Up
FDR	0.433	**0.032**	0.907	**0.018**	0.843	**0.022**	0.638	0.51	0.735
Av. FC	0.062	0.183	-0.013	0.287	-0.006	0.15	0.076	0.044	0.036
N Genes	2	2	2	2	2	2	2	2	2

Tie2 Signaling (R-HSA-210993)

	CORE	UCEC	BRCA	KIRC	KIRP	LUAD	LIHC	STAD	THCA
Regulation	Up	**Up**	Up	Up	Up	Up	Down	Up	Up
FDR	0.741	**0.043**	0.979	0.555	0.934	0.1	0.365	0.746	0.482
Av. FC	0.025	0.13	0.027	0.095	0.003	0.098	-0.017	0.007	0.046
N Genes	2	2	2	2	2	2	2	2	2

Downstream signaling of activated FGFR1 (R-HSA-5654687)

	CORE	UCEC	BRCA	KIRC	KIRP	LUAD	LIHC	STAD	THCA
Regulation	Up	**Up**	Up	Up	Up	Up	Down	Up	Up
FDR	0.741	**0.043**	0.979	0.555	0.934	0.1	0.365	0.746	0.482
Av. FC	0.025	0.13	0.027	0.095	0.003	0.098	-0.017	0.007	0.046
N Genes	2	2	2	2	2	2	2	2	2

Downstream signaling of activated FGFR2 (R-HSA-5654696)

	CORE	UCEC	BRCA	KIRC	KIRP	LUAD	LIHC	STAD	THCA
Regulation	Up	**Up**	Up	Up	Up	Up	Down	Up	Up
FDR	0.741	**0.043**	0.979	0.555	0.934	0.1	0.365	0.746	0.482
Av. FC	0.025	0.13	0.027	0.095	0.003	0.098	-0.017	0.007	0.046
N Genes	2	2	2	2	2	2	2	2	2

Downstream signaling of activated FGFR3 (R-HSA-5654708)

	CORE	UCEC	BRCA	KIRC	KIRP	LUAD	LIHC	STAD	THCA
Regulation	Up	**Up**	Up	Up	Up	Up	Down	Up	Up
FDR	0.741	**0.043**	0.979	0.555	0.934	0.1	0.365	0.746	0.482
Av. FC	0.025	0.13	0.027	0.095	0.003	0.098	-0.017	0.007	0.046
N Genes	2	2	2	2	2	2	2	2	2

RHO GTPases activate PAKs (R-HSA-5627123)

	CORE	UCEC	BRCA	KIRC	KIRP	LUAD	LIHC	STAD	THCA
Regulation	Down	**Down**	Up	Down	Up	Down	Up	Down	Down
FDR	0.15	**0.026**	0.131	0.195	0.854	0.213	0.674	0.868	0.632
Av. FC	-0.434	-0.697	0.313	-0.822	0.032	-0.364	0.092	-0.129	-0.117
N Genes	2	2	2	2	2	2	2	2	2

RHO GTPases Activate ROCKs (R-HSA-5627117)

	CORE	UCEC	BRCA	KIRC	KIRP	LUAD	LIHC	STAD	THCA
Regulation	Down	**Down**	Up	Down	Up	Down	Up	Down	Down
FDR	0.267	**0.028**	0.166	0.1	0.717	0.069	0.999	0.67	0.498
Av. FC	-0.677	-1.249	0.764	-1.457	0.041	-0.658	0.047	-0.279	-0.185
N Genes	1	1	1	1	1	1	1	1	1

HDR through MMEJ (alt-NHEJ) (R-HSA-5685939)

	CORE	UCEC	BRCA	KIRC	KIRP	LUAD	LIHC	STAD	THCA
Regulation	Down	**Down**	Down	**Down**	Down	Down	Up	Up	Up
FDR	0.558	**0.031**	0.421	**0.011**	0.371	0.354	0.835	0.744	0.056
Av. FC	-0.069	-0.049	-0.02	-0.101	-0.004	-0.002	0.001	0.024	0.097
N Genes	3	3	3	3	3	3	3	3	3

ATF4 activates genes in response to endoplasmic reticulum stress (R-HSA-380994)

	CORE	UCEC	BRCA	KIRC	KIRP	LUAD	LIHC	STAD	THCA
Regulation	Up	Up	**Up**	Up	Up	**Up**	Up	Down	Up
FDR	0.416	0.285	**0.025**	0.094	0.261	**0.042**	0.475	0.816	0.354
Av. FC	0.179	0.198	0.413	0.554	0.068	0.45	0.136	-0.027	0.091
N Genes	1	1	1	1	1	1	1	1	1

PERK regulates gene expression (R-HSA-381042)

	CORE	UCEC	BRCA	KIRC	KIRP	LUAD	LIHC	STAD	THCA
Regulation	Up	Up	**Up**	Up	Up	**Up**	Up	Down	Up
FDR	0.416	0.285	**0.025**	0.094	0.261	**0.042**	0.475	0.816	0.354
Av. FC	0.179	0.198	0.413	0.554	0.068	0.45	0.136	-0.027	0.091
N Genes	1	1	1	1	1	1	1	1	1

Aspartate and asparagine metabolism (R-HSA-8963693)

	CORE	UCEC	BRCA	KIRC	KIRP	LUAD	LIHC	STAD	THCA
Regulation	Up	Up	**Up**	Up	Up	**Up**	Up	Down	Up
FDR	0.416	0.285	**0.025**	0.094	0.261	**0.042**	0.475	0.816	0.354
Av. FC	0.179	0.198	0.413	0.554	0.068	0.45	0.136	-0.027	0.091
N Genes	1	1	1	1	1	1	1	1	1

Response of EIF2AK1 (HRI) to heme deficiency (R-HSA-9648895)

	CORE	UCEC	BRCA	KIRC	KIRP	LUAD	LIHC	STAD	THCA
Regulation	Up	Up	**Up**	Up	Up	**Up**	Up	Down	Up
FDR	0.416	0.285	**0.025**	0.094	0.261	**0.042**	0.475	0.816	0.354
Av. FC	0.179	0.198	0.413	0.554	0.068	0.45	0.136	-0.027	0.091
N Genes	1	1	1	1	1	1	1	1	1

E2F-enabled inhibition of pre-replication complex formation (R-HSA-113507)

	CORE	UCEC	BRCA	KIRC	KIRP	LUAD	LIHC	STAD	THCA
Regulation	Up	Up	**Up**	Up	Up	Down	Up	Down	Up
FDR	0.626	0.388	**0.028**	0.411	0.912	0.71	0.073	0.621	0.639
Av. FC	0.063	0.183	0.25	0.173	0.002	-0.047	0.075	-0.076	0.024
N Genes	2	2	2	2	2	2	2	2	2

E2F mediated regulation of DNA replication (R-HSA-113510)

	CORE	UCEC	BRCA	KIRC	KIRP	LUAD	LIHC	STAD	THCA
Regulation	Up	Up	**Up**	Up	Up	Down	Up	Down	Up
FDR	0.626	0.388	**0.028**	0.411	0.912	0.71	0.073	0.621	0.639
Av. FC	0.063	0.183	0.25	0.173	0.002	-0.047	0.075	-0.076	0.024
N Genes	2	2	2	2	2	2	2	2	2

Polo-like kinase mediated events (R-HSA-156711)

	CORE	UCEC	BRCA	KIRC	KIRP	LUAD	LIHC	STAD	THCA
Regulation	Up	Up	Up	**Up**	Down	Up	Up	Down	Down
FDR	0.305	0.09	0.619	**0.003**	0.86	0.454	0.382	0.543	0.846
Av. FC	0.2	0.297	0.167	0.472	-0.012	0.079	0.035	-0.049	-0.001
N Genes	2	2	2	2	2	2	2	2	2

Regulation of FOXO transcriptional activity by acetylation (R-HSA-9617629)

	CORE	UCEC	BRCA	KIRC	KIRP	LUAD	LIHC	STAD	THCA
Regulation	Up	Up	Up	**Up**	Down	Up	Down	Up	Down
FDR	0.524	0.235	0.564	**0.004**	0.81	0.558	0.07	0.403	0.946
Av. FC	0.053	0.067	0.042	0.256	-0.006	0.047	-0.068	0.038	0
N Genes	1	1	1	1	1	1	1	1	1

IRS activation (R-HSA-74713)

	CORE	UCEC	BRCA	KIRC	KIRP	LUAD	LIHC	STAD	THCA
Regulation	Up	Up	Down	**Up**	Down	Up	Up	Up	Up
FDR	0.267	0.09	0.665	**0.01**	0.622	0.106	0.366	0.357	0.916
Av. FC	0.1	0.189	-0.048	0.409	-0.015	0.148	0.12	0.056	0.001
N Genes	1	1	1	1	1	1	1	1	1

Signal attenuation (R-HSA-74749)

	CORE	UCEC	BRCA	KIRC	KIRP	LUAD	LIHC	STAD	THCA
Regulation	Up	Up	Up	**Up**	Down	Up	Up	Up	Up
FDR	0.295	0.106	0.624	**0.01**	0.325	0.238	0.223	0.488	0.762
Av. FC	0.076	0.053	0.03	0.043	-0.065	0.078	0.172	0.01	0.057
N Genes	2	2	2	2	2	2	2	2	2

Cyclin A/B1/B2 associated events during G2/M transition (R-HSA-69273)

	CORE	UCEC	BRCA	KIRC	KIRP	LUAD	LIHC	STAD	THCA
Regulation	Up	Up	Up	**Up**	Down	Up	Up	Down	Up
FDR	0.401	0.143	0.242	**0.01**	0.903	0.407	0.146	0.679	0.806
Av. FC	0.118	0.211	0.164	0.297	-0.005	0.03	0.001	-0.036	0.024
N Genes	3	3	3	3	3	3	3	3	3

AKT phosphorylates targets in the nucleus (R-HSA-198693)

	CORE	UCEC	BRCA	KIRC	KIRP	LUAD	LIHC	STAD	THCA
Regulation	Down	Up	Down	**Up**	Down	Down	Down	Up	Up
FDR	0.478	0.452	0.629	**0.013**	0.764	0.539	0.451	0.619	0.904
Av. FC	-0.053	0.013	-0.028	0.043	-0.029	-0.02	-0.014	0.006	0.03
N Genes	2	2	2	2	2	2	2	2	2

Laminin interactions (R-HSA-3000157)

	CORE	UCEC	BRCA	KIRC	KIRP	LUAD	LIHC	STAD	THCA
Regulation	Up	Down	Down	Up	**Up**	Up	Down	Down	Down
FDR	0.764	0.811	0.532	0.622	**0.007**	0.812	0.678	0.102	0.522
Av. FC	0.017	-0.029	-0.085	0.061	0.119	0.035	-0.065	-0.156	-0.039
N Genes	1	1	1	1	1	1	1	1	1

CHL1 interactions (R-HSA-447041)

	CORE	UCEC	BRCA	KIRC	KIRP	LUAD	LIHC	STAD	THCA
Regulation	Up	Down	Down	Up	**Up**	Up	Down	Down	Down
FDR	0.764	0.811	0.532	0.622	**0.007**	0.812	0.678	0.102	0.522
Av. FC	0.017	-0.029	-0.085	0.061	0.119	0.035	-0.065	-0.156	-0.039
N Genes	1	1	1	1	1	1	1	1	1

Platelet Adhesion to exposed collagen (R-HSA-75892)

	CORE	UCEC	BRCA	KIRC	KIRP	LUAD	LIHC	STAD	THCA
Regulation	Up	Down	Down	Up	**Up**	Up	Down	Down	Down
FDR	0.764	0.811	0.532	0.622	**0.007**	0.812	0.678	0.102	0.522
Av. FC	0.017	-0.029	-0.085	0.061	0.119	0.035	-0.065	-0.156	-0.039
N Genes	1	1	1	1	1	1	1	1	1

L1CAM interactions (R-HSA-373760)

	CORE	UCEC	BRCA	KIRC	KIRP	LUAD	LIHC	STAD	THCA
Regulation	Down	Up	Up	Down	**Up**	Down	Up	Down	Up
FDR	0.859	0.922	0.513	0.908	**0.017**	0.978	0.905	0.377	0.869
Av. FC	-0.005	0.015	0.068	-0.06	0.02	-0.006	0.072	-0.011	0.008
N Genes	5	5	5	5	5	5	5	5	5

Uptake and actions of bacterial toxins (R-HSA-5339562)

	CORE	UCEC	BRCA	KIRC	KIRP	LUAD	LIHC	STAD	THCA
Regulation	Down	Down	Up	Down	**Up**	Down	Up	Down	Up
FDR	0.477	0.078	0.537	0.642	**0.02**	0.909	0.816	0.93	0.189
Av. FC	-0.134	-0.097	0.016	-0.139	0.002	-0.037	0.101	-0.009	0.054
N Genes	2	2	2	2	2	2	2	2	2

Signaling by MAP2K mutants (R-HSA-9652169)

	CORE	UCEC	BRCA	KIRC	KIRP	LUAD	LIHC	STAD	THCA
Regulation	Up	Up	Up	Down	**Up**	Down	Up	Down	Up
FDR	0.728	0.344	0.322	0.541	**0.049**	0.865	0.579	0.831	0.409
Av. FC	0.033	0.04	0.11	-0.192	0.006	-0.034	0.13	-0.028	0.031
N Genes	2	2	2	2	2	2	2	2	2

RUNX1 interacts with co-factors whose precise effect on RUNX1 targets is not known (R-HSA-8939243)

	CORE	UCEC	BRCA	KIRC	KIRP	LUAD	LIHC	STAD	THCA
Regulation	Up	Up	Up	Up	**Down**	Up	Down	Down	**Down**
FDR	0.648	0.054	0.919	0.208	**0.009**	0.911	0.992	0.172	**0.009**
Av. FC	0.021	0.175	0.008	0.09	0	0.007	0	0	-0.081
N Genes	1	1	1	1	1	1	1	1	1

RHOBTB3 ATPase cycle (R-HSA-9706019)

	CORE	UCEC	BRCA	KIRC	KIRP	LUAD	LIHC	STAD	THCA
Regulation	Down	Up	Down	Down	**Down**	Up	Up	Up	Down
FDR	0.266	0.261	0.503	0.651	**0.01**	0.941	0.898	0.89	0.868
Av. FC	-0.258	0.097	-0.203	-0.355	-0.217	0.034	0.083	0.059	-0.023
N Genes	2	2	2	2	2	2	2	2	2

RHOBTB GTPase Cycle (R-HSA-9706574)

	CORE	UCEC	BRCA	KIRC	KIRP	LUAD	LIHC	STAD	THCA
Regulation	Down	Up	Down	Down	**Down**	Up	Up	Up	Down
FDR	0.266	0.261	0.503	0.651	**0.01**	0.941	0.898	0.89	0.868
Av. FC	-0.258	0.097	-0.203	-0.355	-0.217	0.034	0.083	0.059	-0.023
N Genes	2	2	2	2	2	2	2	2	2

MET activates PTK2 signaling (R-HSA-8874081)

	CORE	UCEC	BRCA	KIRC	KIRP	LUAD	LIHC	STAD	THCA
Regulation	Down	Down	Up	Down	**Down**	Up	Up	Down	Up
FDR	0.788	0.958	0.698	0.786	**0.024**	0.938	0.843	0.098	0.664
Av. FC	-0.074	-0.015	0.043	-0.025	-0.003	0.043	0.069	-0.043	0.08
N Genes	3	3	3	3	3	3	3	3	3

MET promotes cell motility (R-HSA-8875878)

	CORE	UCEC	BRCA	KIRC	KIRP	LUAD	LIHC	STAD	THCA
Regulation	Down	Down	Up	Down	**Down**	Up	Up	Down	Up
FDR	0.788	0.958	0.698	0.786	**0.024**	0.938	0.843	0.098	0.664
Av. FC	-0.074	-0.015	0.043	-0.025	-0.003	0.043	0.069	-0.043	0.08
N Genes	3	3	3	3	3	3	3	3	3

Metabolism of vitamins and cofactors (R-HSA-196854)

	CORE	UCEC	BRCA	KIRC	KIRP	LUAD	LIHC	STAD	THCA
Regulation	Up	Up	Down	Up	**Down**	Up	Up	Down	Up
FDR	0.081	0.187	0.235	0.42	**0.025**	0.387	0.887	0.907	0.673
Av. FC	0.206	0.178	-0.314	0.062	-0.109	0.103	0.134	-0.023	0.02
N Genes	3	3	3	3	3	3	3	3	3

ChREBP activates metabolic gene expression (R-HSA-163765)

	CORE	UCEC	BRCA	KIRC	KIRP	LUAD	LIHC	STAD	THCA
Regulation	Up	Up	Down	Up	**Down**	Up	Up	Down	Down
FDR	0.087	0.117	0.19	0.502	**0.025**	0.356	0.675	0.862	0.58
Av. FC	0.388	0.288	-0.422	0.179	-0.138	0.198	0.182	-0.023	0
N Genes	2	2	2	2	2	2	2	2	2

Metabolism of water-soluble vitamins and cofactors (R-HSA-196849)

	CORE	UCEC	BRCA	KIRC	KIRP	LUAD	LIHC	STAD	THCA
Regulation	Up	Up	Down	Up	**Down**	Up	Up	Down	Down
FDR	0.087	0.117	0.19	0.502	**0.025**	0.356	0.675	0.862	0.58
Av. FC	0.388	0.288	-0.422	0.179	-0.138	0.198	0.182	-0.023	0
N Genes	2	2	2	2	2	2	2	2	2

Defective Mismatch Repair Associated With MSH3 (R-HSA-5632927)

	CORE	UCEC	BRCA	KIRC	KIRP	LUAD	LIHC	STAD	THCA
Regulation	Up	Up	Down	Up	**Down**	Up	Down	Down	Down
FDR	0.143	0.185	0.639	0.079	**0.026**	0.871	0.261	0.608	0.769
Av. FC	0.234	0.176	-0.07	0.265	-0.129	0.027	-0.162	-0.041	-0.013
N Genes	1	1	1	1	1	1	1	1	1

PTK6 Regulates Cell Cycle (R-HSA-8849470)

	CORE	UCEC	BRCA	KIRC	KIRP	LUAD	LIHC	STAD	THCA
Regulation	Up	Up	Up	Down	**Down**	Up	Down	Up	Up
FDR	0.772	0.582	0.669	0.982	**0.028**	0.879	0.989	0.961	0.745
Av. FC	0.028	0.138	0.064	-0.003	-0.008	0.011	-0.049	0.003	0.007
N Genes	3	3	3	3	3	3	3	3	3

Rho GTPase cycle (R-HSA-194840)

	CORE	UCEC	BRCA	KIRC	KIRP	LUAD	LIHC	STAD	THCA
Regulation	Down	Up	Down	Down	**Down**	Up	Up	Up	Down
FDR	0.282	0.104	0.405	0.858	**0.031**	0.451	0.975	0.963	0.735
Av. FC	-0.127	0.145	-0.23	-0.229	-0.124	0.115	0.045	0.038	-0.057
N Genes	3	3	3	3	3	3	3	3	3

Vitamin B5 (pantothenate) metabolism (R-HSA-199220)

	CORE	UCEC	BRCA	KIRC	KIRP	LUAD	LIHC	STAD	THCA
Regulation	Up	Up	Down	Up	**Down**	Up	Up	Up	Up
FDR	0.222	0.149	0.094	0.46	**0.036**	0.293	0.985	0.873	0.474
Av. FC	0.319	0.311	-0.651	0.157	-0.142	0.259	0.071	0.018	0.045
N Genes	1	1	1	1	1	1	1	1	1

Gamma carboxylation, hypusine formation and arylsulfatase activation (R-HSA-163841)

	CORE	UCEC	BRCA	KIRC	KIRP	LUAD	LIHC	STAD	THCA
Regulation	Down	Down	Down	Down	**Down**	Up	Up	Up	Up
FDR	0.221	0.051	0.606	0.403	**0.039**	0.971	0.566	0.922	0.085
Av. FC	-0.281	-0.359	-0.081	-0.217	-0.123	0.002	0.165	0.001	0.159
N Genes	1	1	1	1	1	1	1	1	1

Uptake and function of diphtheria toxin (R-HSA-5336415)

	CORE	UCEC	BRCA	KIRC	KIRP	LUAD	LIHC	STAD	THCA
Regulation	Down	Down	Down	Down	**Down**	Up	Up	Up	Up
FDR	0.221	0.051	0.606	0.403	**0.039**	0.971	0.566	0.922	0.085
Av. FC	-0.281	-0.359	-0.081	-0.217	-0.123	0.002	0.165	0.001	0.159
N Genes	1	1	1	1	1	1	1	1	1

Synthesis of diphthamide-EEF2 (R-HSA-5358493)

	CORE	UCEC	BRCA	KIRC	KIRP	LUAD	LIHC	STAD	THCA
Regulation	Down	Down	Down	Down	**Down**	Up	Up	Up	Up
FDR	0.221	0.051	0.606	0.403	**0.039**	0.971	0.566	0.922	0.085
Av. FC	-0.281	-0.359	-0.081	-0.217	-0.123	0.002	0.165	0.001	0.159
N Genes	1	1	1	1	1	1	1	1	1

Protein methylation (R-HSA-8876725)

	CORE	UCEC	BRCA	KIRC	KIRP	LUAD	LIHC	STAD	THCA
Regulation	Down	Down	Down	Down	**Down**	Up	Up	Up	Up
FDR	0.221	0.051	0.606	0.403	**0.039**	0.971	0.566	0.922	0.085
Av. FC	-0.281	-0.359	-0.081	-0.217	-0.123	0.002	0.165	0.001	0.159
N Genes	1	1	1	1	1	1	1	1	1

Diseases of metabolism (R-HSA-5668914)

	CORE	UCEC	BRCA	KIRC	KIRP	LUAD	LIHC	STAD	THCA
Regulation	Up	Up	Down	Up	**Down**	Up	Up	Down	Down
FDR	0.173	0.077	0.352	0.519	**0.041**	0.124	0.408	0.474	0.889
Av. FC	0.243	0.187	-0.043	0.142	-0.042	0.141	0.12	-0.003	-0.021
N Genes	2	2	2	2	2	2	2	2	2

ECM proteoglycans (R-HSA-3000178)

	CORE	UCEC	BRCA	KIRC	KIRP	LUAD	LIHC	STAD	THCA
Regulation	Down	Up	Up	Down	**Down**	Up	Up	Down	Down
FDR	0.79	0.897	0.912	0.884	**0.041**	0.892	0.686	0.342	0.141
Av. FC	-0.102	0.006	0.015	-0.027	-0.017	0.09	0.137	-0.105	-0.003
N Genes	3	3	3	3	3	3	3	3	3

TP53 Regulates Transcription of Cell Death Genes (R-HSA-5633008)

	CORE	UCEC	BRCA	KIRC	KIRP	LUAD	LIHC	STAD	THCA
Regulation	Up	Up	Up	Up	Down	**Up**	Up	Up	**Up**
FDR	0.574	0.707	0.249	0.082	0.509	**0.002**	0.474	0.296	**0.017**
Av. FC	0.039	0.07	0.145	0.092	-0.01	0.149	0.048	0.052	0.085
N Genes	4	4	4	4	4	4	4	4	4

TP53 Regulates Transcription of Genes Involved in Cytochrome C Release (R-HSA-6803204)

	CORE	UCEC	BRCA	KIRC	KIRP	LUAD	LIHC	STAD	THCA
Regulation	Up	Up	Up	Up	Down	**Up**	Up	Up	**Up**
FDR	0.574	0.707	0.249	0.082	0.509	**0.002**	0.474	0.296	**0.017**
Av. FC	0.039	0.07	0.145	0.092	-0.01	0.149	0.048	0.052	0.085
N Genes	4	4	4	4	4	4	4	4	4

Unfolded Protein Response (UPR) (R-HSA-381119)

	CORE	UCEC	BRCA	KIRC	KIRP	LUAD	LIHC	STAD	THCA
Regulation	Up	Up	Up	Up	Up	**Up**	Up	Down	Up
FDR	0.591	0.186	0.084	0.062	0.27	**0.005**	0.57	0.472	0.15
Av. FC	0.073	0.048	0.236	0.404	0.013	0.355	0.037	-0.076	0.094
N Genes	2	2	2	2	2	2	2	2	2

Autodegradation of the E3 ubiquitin ligase COP1 (R-HSA-349425)

	CORE	UCEC	BRCA	KIRC	KIRP	LUAD	LIHC	STAD	THCA
Regulation	Up	Up	Up	Up	Up	**Up**	Up	Up	Up
FDR	0.557	0.725	0.237	0.252	0.858	**0.008**	0.082	0.232	0.815
Av. FC	0.119	0.094	0.206	0.158	0.009	0.312	0.047	0.094	0.028
N Genes	2	2	2	2	2	2	2	2	2

TP53 Regulates Transcription of Caspase Activators and Caspases (R-HSA-6803207)

	CORE	UCEC	BRCA	KIRC	KIRP	LUAD	LIHC	STAD	THCA
Regulation	Up	Up	Up	Up	Up	**Up**	Up	Up	Up
FDR	0.557	0.725	0.237	0.252	0.858	**0.008**	0.082	0.232	0.815
Av. FC	0.119	0.094	0.206	0.158	0.009	0.312	0.047	0.094	0.028
N Genes	2	2	2	2	2	2	2	2	2

Pexophagy (R-HSA-9664873)

	CORE	UCEC	BRCA	KIRC	KIRP	LUAD	LIHC	STAD	THCA
Regulation	Up	Down	Up	Down	Down	**Up**	Up	Up	Up
FDR	0.26	0.909	0.064	0.713	0.781	**0.016**	0.185	0.065	0.747
Av. FC	0.266	-0.019	0.361	-0.09	-0.01	0.348	0.292	0.214	0.023
N Genes	1	1	1	1	1	1	1	1	1

Diseases of Mismatch Repair (MMR) (R-HSA-5423599)

	CORE	UCEC	BRCA	KIRC	KIRP	LUAD	LIHC	STAD	THCA
Regulation	Up	Up	Up	Up	Down	Up	**Up**	Up	Down
FDR	0.422	0.31	0.675	0.212	0.244	0.978	**0.006**	0.515	0.513
Av. FC	0.118	0.143	0.017	0.174	-0.065	0.008	0.062	0.022	-0.035
N Genes	2	2	2	2	2	2	2	2	2

Defective Mismatch Repair Associated With MSH2 (R-HSA-5632928)

	CORE	UCEC	BRCA	KIRC	KIRP	LUAD	LIHC	STAD	THCA
Regulation	Up	Up	Up	Up	Down	Up	**Up**	Up	Down
FDR	0.422	0.31	0.675	0.212	0.244	0.978	**0.006**	0.515	0.513
Av. FC	0.118	0.143	0.017	0.174	-0.065	0.008	0.062	0.022	-0.035
N Genes	2	2	2	2	2	2	2	2	2

Defective Mismatch Repair Associated With MSH6 (R-HSA-5632968)

	CORE	UCEC	BRCA	KIRC	KIRP	LUAD	LIHC	STAD	THCA
Regulation	Up	Up	Up	Up	Down	Up	**Up**	Up	Down
FDR	0.422	0.31	0.675	0.212	0.244	0.978	**0.006**	0.515	0.513
Av. FC	0.118	0.143	0.017	0.174	-0.065	0.008	0.062	0.022	-0.035
N Genes	2	2	2	2	2	2	2	2	2

Diseases of DNA repair (R-HSA-9675135)

	CORE	UCEC	BRCA	KIRC	KIRP	LUAD	LIHC	STAD	THCA
Regulation	Up	Up	Up	Up	Down	Up	**Up**	Up	Down
FDR	0.422	0.31	0.675	0.212	0.244	0.978	**0.006**	0.515	0.513
Av. FC	0.118	0.143	0.017	0.174	-0.065	0.008	0.062	0.022	-0.035
N Genes	2	2	2	2	2	2	2	2	2

HIV Life Cycle (R-HSA-162587)

	CORE	UCEC	BRCA	KIRC	KIRP	LUAD	LIHC	STAD	THCA
Regulation	Up	Down	Up	Up	Down	Up	**Up**	Up	Down
FDR	0.413	0.877	0.244	0.6	0.721	0.625	**0.008**	0.934	0.312
Av. FC	0.15	-0.016	0.139	0.09	-0.012	0.045	0.214	0.001	-0.085
N Genes	1	1	1	1	1	1	1	1	1

Integration of provirus (R-HSA-162592)

	CORE	UCEC	BRCA	KIRC	KIRP	LUAD	LIHC	STAD	THCA
Regulation	Up	Down	Up	Up	Down	Up	**Up**	Up	Down
FDR	0.413	0.877	0.244	0.6	0.721	0.625	**0.008**	0.934	0.312
Av. FC	0.15	-0.016	0.139	0.09	-0.012	0.045	0.214	0.001	-0.085
N Genes	1	1	1	1	1	1	1	1	1

Early Phase of HIV Life Cycle (R-HSA-162594)

	CORE	UCEC	BRCA	KIRC	KIRP	LUAD	LIHC	STAD	THCA
Regulation	Up	Down	Up	Up	Down	Up	**Up**	Up	Down
FDR	0.413	0.877	0.244	0.6	0.721	0.625	**0.008**	0.934	0.312
Av. FC	0.15	-0.016	0.139	0.09	-0.012	0.045	0.214	0.001	-0.085
N Genes	1	1	1	1	1	1	1	1	1

2-LTR circle formation (R-HSA-164843)

	CORE	UCEC	BRCA	KIRC	KIRP	LUAD	LIHC	STAD	THCA
Regulation	Up	Down	Up	Up	Down	Up	**Up**	Up	Down
FDR	0.413	0.877	0.244	0.6	0.721	0.625	**0.008**	0.934	0.312
Av. FC	0.15	-0.016	0.139	0.09	-0.012	0.045	0.214	0.001	-0.085
N Genes	1	1	1	1	1	1	1	1	1

CD209 (DC-SIGN) signaling (R-HSA-5621575)

	CORE	UCEC	BRCA	KIRC	KIRP	LUAD	LIHC	STAD	THCA
Regulation	Down	Up	Up	Up	Down	Up	**Up**	Up	Up
FDR	0.646	0.521	0.512	0.342	0.875	0.629	**0.008**	0.136	0.951
Av. FC	-0.012	0.055	0.053	0.072	-0.002	0.003	0.023	0.025	0.009
N Genes	2	2	2	2	2	2	2	2	2

Cellular response to heat stress (R-HSA-3371556)

	CORE	UCEC	BRCA	KIRC	KIRP	LUAD	LIHC	STAD	THCA
Regulation	Down	Down	Up	Down	Down	Up	**Up**	**Up**	Up
FDR	0.51	0.915	0.443	0.942	0.27	0.421	**0.012**	**0.042**	0.223
Av. FC	-0.022	-0.013	0.105	-0.073	-0.022	0.081	0.17	0.059	0.095
N Genes	6	6	6	6	6	6	6	6	6

M Phase (R-HSA-68886)

	CORE	UCEC	BRCA	KIRC	KIRP	LUAD	LIHC	STAD	THCA
Regulation	Up	Up	Up	Up	Down	Down	**Up**	Down	Up
FDR	0.763	0.88	0.408	0.646	0.864	0.882	**0.012**	0.621	0.217
Av. FC	0.051	0.052	0.12	0.009	-0.026	-0.01	0.096	-0.048	0.023
N Genes	5	5	5	5	5	5	5	5	5

Regulation of HSF1-mediated heat shock response (R-HSA-3371453)

	CORE	UCEC	BRCA	KIRC	KIRP	LUAD	LIHC	STAD	THCA
Regulation	Down	Down	Up	Down	Up	Up	**Up**	**Up**	Up
FDR	0.354	0.978	0.254	0.832	0.392	0.251	**0.013**	**0.026**	0.06
Av. FC	-0.016	0	0.186	-0.082	0.006	0.093	0.188	0.084	0.158
N Genes	4	4	4	4	4	4	4	4	4

Signaling by TGFB family members (R-HSA-9006936)

	CORE	UCEC	BRCA	KIRC	KIRP	LUAD	LIHC	STAD	THCA
Regulation	Down	Down	Up	Down	Down	Up	**Up**	Down	Down
FDR	0.561	0.864	0.761	0.978	0.988	0.26	**0.014**	0.446	0.436
Av. FC	-0.103	-0.008	0.064	-0.029	-0.01	0.021	0.066	-0.043	-0.016
N Genes	6	6	6	6	6	6	6	6	6

CREB1 phosphorylation through NMDA receptor-mediated activation of RAS signaling (R-HSA-442742)

	CORE	UCEC	BRCA	KIRC	KIRP	LUAD	LIHC	STAD	THCA
Regulation	Up	Down	Up	Down	Down	Up	**Up**	Down	Up
FDR	0.603	0.221	0.702	0.528	0.28	0.896	**0.014**	0.845	0.457
Av. FC	0.039	0	0.069	-0.149	-0.056	0.026	0.078	-0.028	0.067
N Genes	2	2	2	2	2	2	2	2	2

Estrogen-stimulated signaling through PRKCZ (R-HSA-9634635)

	CORE	UCEC	BRCA	KIRC	KIRP	LUAD	LIHC	STAD	THCA
Regulation	Up	Down	Up	Down	Down	Up	**Up**	Down	Up
FDR	0.603	0.221	0.702	0.528	0.28	0.896	**0.014**	0.845	0.457
Av. FC	0.039	0	0.069	-0.149	-0.056	0.026	0.078	-0.028	0.067
N Genes	2	2	2	2	2	2	2	2	2

G2/M DNA damage checkpoint (R-HSA-69473)

	CORE	UCEC	BRCA	KIRC	KIRP	LUAD	LIHC	STAD	THCA
Regulation	Down	Up	Up	Up	Up	Up	**Up**	Up	Up
FDR	0.825	0.819	0.39	0.33	0.831	0.225	**0.018**	0.201	0.471
Av. FC	-0.016	0.03	0.064	0.047	0.003	0.044	0.04	0.015	0.032
N Genes	12	12	12	12	12	12	12	12	12

G2/M Checkpoints (R-HSA-69481)

	CORE	UCEC	BRCA	KIRC	KIRP	LUAD	LIHC	STAD	THCA
Regulation	Down	Up	Up	Up	Up	Up	**Up**	Up	Up
FDR	0.825	0.819	0.39	0.33	0.831	0.225	**0.018**	0.201	0.471
Av. FC	-0.016	0.03	0.064	0.047	0.003	0.044	0.04	0.015	0.032
N Genes	12	12	12	12	12	12	12	12	12

Signaling by BMP (R-HSA-201451)

	CORE	UCEC	BRCA	KIRC	KIRP	LUAD	LIHC	STAD	THCA
Regulation	Down	Up	Up	Down	Up	Down	**Up**	Down	Up
FDR	0.593	0.947	0.623	0.949	0.956	0.14	**0.019**	0.589	0.909
Av. FC	-0.049	0.019	0.043	-0.015	0.004	-0.009	0.002	-0.011	0.01
N Genes	3	3	3	3	3	3	3	3	3

Rap1 signalling (R-HSA-392517)

	CORE	UCEC	BRCA	KIRC	KIRP	LUAD	LIHC	STAD	THCA
Regulation	Down	Up	Up	Down	Down	Down	**Up**	Up	Up
FDR	0.574	0.982	0.641	0.457	0.834	0.469	**0.023**	0.61	0.998
Av. FC	-0.072	0.015	0.055	-0.009	-0.006	-0.035	0.06	0.021	0.001
N Genes	3	3	3	3	3	3	3	3	3

Mismatch Repair (R-HSA-5358508)

	CORE	UCEC	BRCA	KIRC	KIRP	LUAD	LIHC	STAD	THCA
Regulation	Up	Up	Up	Up	Down	Up	**Up**	Down	Up
FDR	0.539	0.399	0.582	0.387	0.38	0.877	**0.024**	0.431	0.23
Av. FC	0.057	0.113	0.045	0.118	-0.047	0.037	0.058	-0.018	0.003
N Genes	3	3	3	3	3	3	3	3	3

RUNX3 regulates CDKN1A transcription (R-HSA-8941855)

	CORE	UCEC	BRCA	KIRC	KIRP	LUAD	LIHC	STAD	THCA
Regulation	Down	Up	Down	Up	Down	Up	**Down**	**Down**	Up
FDR	0.239	0.428	0.337	0.277	0.938	0.083	**0.038**	**0.038**	0.688
Av. FC	-0.111	0.005	-0.011	0.076	-0.003	0.009	-0.051	-0.023	0.034
N Genes	4	4	4	4	4	4	4	4	4

Loss of Nlp from mitotic centrosomes (R-HSA-380259)

	CORE	UCEC	BRCA	KIRC	KIRP	LUAD	LIHC	STAD	THCA
Regulation	Down	Up	Up	Up	Up	Down	**Down**	Down	Up
FDR	0.668	0.9	0.235	0.637	0.843	0.233	**0.047**	0.303	0.239
Av. FC	-0.038	0.021	0.074	0.001	0.01	0	-0.059	-0.038	0.078
N Genes	2	2	2	2	2	2	2	2	2

Recruitment of mitotic centrosome proteins and complexes (R-HSA-380270)

	CORE	UCEC	BRCA	KIRC	KIRP	LUAD	LIHC	STAD	THCA
Regulation	Down	Up	Up	Up	Up	Down	**Down**	Down	Up
FDR	0.668	0.9	0.235	0.637	0.843	0.233	**0.047**	0.303	0.239
Av. FC	-0.038	0.021	0.074	0.001	0.01	0	-0.059	-0.038	0.078
N Genes	2	2	2	2	2	2	2	2	2

Loss of proteins required for interphase microtubule organization from the centrosome (R-HSA-380284)

	CORE	UCEC	BRCA	KIRC	KIRP	LUAD	LIHC	STAD	THCA
Regulation	Down	Up	Up	Up	Up	Down	**Down**	Down	Up
FDR	0.668	0.9	0.235	0.637	0.843	0.233	**0.047**	0.303	0.239
Av. FC	-0.038	0.021	0.074	0.001	0.01	0	-0.059	-0.038	0.078
N Genes	2	2	2	2	2	2	2	2	2

Centrosome maturation (R-HSA-380287)

	CORE	UCEC	BRCA	KIRC	KIRP	LUAD	LIHC	STAD	THCA
Regulation	Down	Up	Up	Up	Up	Down	**Down**	Down	Up
FDR	0.668	0.9	0.235	0.637	0.843	0.233	**0.047**	0.303	0.239
Av. FC	-0.038	0.021	0.074	0.001	0.01	0	-0.059	-0.038	0.078
N Genes	2	2	2	2	2	2	2	2	2

Recruitment of NuMA to mitotic centrosomes (R-HSA-380320)

	CORE	UCEC	BRCA	KIRC	KIRP	LUAD	LIHC	STAD	THCA
Regulation	Down	Up	Up	Up	Up	Down	**Down**	Down	Up
FDR	0.668	0.9	0.235	0.637	0.843	0.233	**0.047**	0.303	0.239
Av. FC	-0.038	0.021	0.074	0.001	0.01	0	-0.059	-0.038	0.078
N Genes	2	2	2	2	2	2	2	2	2

AURKA Activation by TPX2 (R-HSA-8854518)

	CORE	UCEC	BRCA	KIRC	KIRP	LUAD	LIHC	STAD	THCA
Regulation	Down	Up	Up	Up	Up	Down	**Down**	Down	Up
FDR	0.668	0.9	0.235	0.637	0.843	0.233	**0.047**	0.303	0.239
Av. FC	-0.038	0.021	0.074	0.001	0.01	0	-0.059	-0.038	0.078
N Genes	2	2	2	2	2	2	2	2	2

RNA Polymerase II Transcription (R-HSA-73857)

	CORE	UCEC	BRCA	KIRC	KIRP	LUAD	LIHC	STAD	THCA
Regulation	Down	Up	Up	Up	Down	Up	Up	**Up**	Up
FDR	0.405	0.75	0.106	0.262	0.837	0.143	0.232	**0.003**	0.14
Av. FC	-0.029	0.016	0.025	0.02	-0.006	0.024	0.035	0.003	0.007
N Genes	55	55	55	55	55	55	55	55	55

Gene expression (Transcription) (R-HSA-74160)

	CORE	UCEC	BRCA	KIRC	KIRP	LUAD	LIHC	STAD	THCA
Regulation	Down	Up	Up	Up	Down	Up	Up	**Up**	Up
FDR	0.405	0.75	0.106	0.262	0.837	0.143	0.232	**0.003**	0.14
Av. FC	-0.029	0.016	0.025	0.02	-0.006	0.024	0.035	0.003	0.007
N Genes	55	55	55	55	55	55	55	55	55

Generic Transcription Pathway (R-HSA-212436)

	CORE	UCEC	BRCA	KIRC	KIRP	LUAD	LIHC	STAD	THCA
Regulation	Down	Up	Up	Up	Down	Up	Up	**Up**	Up
FDR	0.354	0.716	0.116	0.239	0.804	0.185	0.336	**0.004**	0.144
Av. FC	-0.029	0.016	0.027	0.019	-0.006	0.023	0.037	0.003	0.008
N Genes	54	54	54	54	54	54	54	54	54

Selective autophagy (R-HSA-9663891)

	CORE	UCEC	BRCA	KIRC	KIRP	LUAD	LIHC	STAD	THCA
Regulation	Up	Up	Up	Down	Down	Up	Up	**Up**	Up
FDR	0.601	0.965	0.116	0.711	0.898	0.311	0.549	**0.004**	0.805
Av. FC	0.084	0.017	0.134	-0.055	-0.019	0.13	0.119	0.071	0.025
N Genes	3	3	3	3	3	3	3	3	3

Ubiquitin Mediated Degradation of Phosphorylated Cdc25A (R-HSA-69601)

	CORE	UCEC	BRCA	KIRC	KIRP	LUAD	LIHC	STAD	THCA
Regulation	Down	Down	Down	Up	Down	Down	Up	**Up**	Up
FDR	0.135	0.888	0.49	0.679	0.299	0.637	0.512	**0.012**	0.301
Av. FC	-0.189	-0.034	-0.078	0.017	-0.018	-0.065	0.036	0.007	0.014
N Genes	2	2	2	2	2	2	2	2	2

p53-Independent DNA Damage Response (R-HSA-69610)

	CORE	UCEC	BRCA	KIRC	KIRP	LUAD	LIHC	STAD	THCA
Regulation	Down	Down	Down	Up	Down	Down	Up	**Up**	Up
FDR	0.135	0.888	0.49	0.679	0.299	0.637	0.512	**0.012**	0.301
Av. FC	-0.189	-0.034	-0.078	0.017	-0.018	-0.065	0.036	0.007	0.014
N Genes	2	2	2	2	2	2	2	2	2

p53-Independent G1/S DNA damage checkpoint (R-HSA-69613)

	CORE	UCEC	BRCA	KIRC	KIRP	LUAD	LIHC	STAD	THCA
Regulation	Down	Down	Down	Up	Down	Down	Up	**Up**	Up
FDR	0.135	0.888	0.49	0.679	0.299	0.637	0.512	**0.012**	0.301
Av. FC	-0.189	-0.034	-0.078	0.017	-0.018	-0.065	0.036	0.007	0.014
N Genes	2	2	2	2	2	2	2	2	2

Activation of ATR in response to replication stress (R-HSA-176187)

	CORE	UCEC	BRCA	KIRC	KIRP	LUAD	LIHC	STAD	THCA
Regulation	Down	Down	Down	Down	Up	Down	Down	**Up**	Up
FDR	0.055	0.933	0.167	0.54	0.556	0.279	0.545	**0.023**	0.1
Av. FC	-0.286	-0.004	-0.121	-0.047	0.013	-0.105	-0.051	0.159	0.056
N Genes	1	1	1	1	1	1	1	1	1

Ub-specific processing proteases (R-HSA-5689880)

	CORE	UCEC	BRCA	KIRC	KIRP	LUAD	LIHC	STAD	THCA
Regulation	Down	Up	Down	Up	Up	Up	Down	**Up**	Up
FDR	0.165	0.615	0.441	0.261	0.907	0.553	0.419	**0.039**	0.888
Av. FC	-0.041	0.03	-0.084	0.063	0.021	0.012	-0.008	0.022	0.006
N Genes	10	10	10	10	10	10	10	10	10

Processing of DNA double-strand break ends (R-HSA-5693607)

	CORE	UCEC	BRCA	KIRC	KIRP	LUAD	LIHC	STAD	THCA
Regulation	Down	Down	Up	Down	Up	Up	Up	**Up**	Up
FDR	0.519	0.351	0.138	0.201	0.493	0.168	0.352	**0.042**	0.063
Av. FC	0	-0.036	0.034	-0.036	0.011	0.073	0.079	0.114	0.048
N Genes	5	5	5	5	5	5	5	5	5

Macroautophagy (R-HSA-1632852)

	CORE	UCEC	BRCA	KIRC	KIRP	LUAD	LIHC	STAD	THCA
Regulation	Up	Up	Up	Down	Down	Up	**Up**	**Up**	Down
FDR	0.925	0.511	0.266	0.739	0.892	0.372	**0.049**	**0.048**	0.491
Av. FC	0.043	0.006	0.053	-0.031	-0.022	0.072	0.111	0.049	-0.016
N Genes	9	9	9	9	9	9	9	9	9

Autophagy (R-HSA-9612973)

	CORE	UCEC	BRCA	KIRC	KIRP	LUAD	LIHC	STAD	THCA
Regulation	Up	Up	Up	Down	Down	Up	**Up**	**Up**	Down
FDR	0.925	0.511	0.266	0.739	0.892	0.372	**0.049**	**0.048**	0.491
Av. FC	0.043	0.006	0.053	-0.031	-0.022	0.072	0.111	0.049	-0.016
N Genes	9	9	9	9	9	9	9	9	9

Detoxification of Reactive Oxygen Species (R-HSA-3299685)

	CORE	UCEC	BRCA	KIRC	KIRP	LUAD	LIHC	STAD	THCA
Regulation	**Down**	Up	Down	Down	Down	Up	Down	**Down**	Up
FDR	**0.04**	0.922	0.858	0.264	0.226	0.305	0.482	**0.01**	0.128
Av. FC	-0.208	0.006	-0.025	-0.188	-0.063	0.134	-0.088	-0.167	0.11
N Genes	1	1	1	1	1	1	1	1	1

Signaling by Activin (R-HSA-1502540)

	CORE	UCEC	BRCA	KIRC	KIRP	LUAD	LIHC	STAD	THCA
Regulation	Down	Down	Up	Down	Down	Down	Up	**Down**	Up
FDR	0.526	0.77	0.474	0.476	0.967	0.42	0.084	**0.012**	0.473
Av. FC	-0.057	-0.031	0.048	-0.101	-0.002	-0.042	0.056	-0.096	0.039
N Genes	2	2	2	2	2	2	2	2	2

TGF-beta receptor signaling activates SMADs (R-HSA-2173789)

	CORE	UCEC	BRCA	KIRC	KIRP	LUAD	LIHC	STAD	THCA
Regulation	Down	Down	Up	Down	Down	Down	Up	**Down**	Up
FDR	0.526	0.77	0.474	0.476	0.967	0.42	0.084	**0.012**	0.473
Av. FC	-0.057	-0.031	0.048	-0.101	-0.002	-0.042	0.056	-0.096	0.039
N Genes	2	2	2	2	2	2	2	2	2

Downregulation of SMAD2/3:SMAD4 transcriptional activity (R-HSA-2173795)

	CORE	UCEC	BRCA	KIRC	KIRP	LUAD	LIHC	STAD	THCA
Regulation	Down	Down	Up	Down	Down	Down	Up	**Down**	Up
FDR	0.526	0.77	0.474	0.476	0.967	0.42	0.084	**0.012**	0.473
Av. FC	-0.057	-0.031	0.048	-0.101	-0.002	-0.042	0.056	-0.096	0.039
N Genes	2	2	2	2	2	2	2	2	2

Loss of Function of SMAD4 in Cancer (R-HSA-3304347)

	CORE	UCEC	BRCA	KIRC	KIRP	LUAD	LIHC	STAD	THCA
Regulation	Down	Down	Up	Down	Down	Down	Up	**Down**	Up
FDR	0.526	0.77	0.474	0.476	0.967	0.42	0.084	**0.012**	0.473
Av. FC	-0.057	-0.031	0.048	-0.101	-0.002	-0.042	0.056	-0.096	0.039
N Genes	2	2	2	2	2	2	2	2	2

Loss of Function of SMAD2/3 in Cancer (R-HSA-3304349)

	CORE	UCEC	BRCA	KIRC	KIRP	LUAD	LIHC	STAD	THCA
Regulation	Down	Down	Up	Down	Down	Down	Up	**Down**	Up
FDR	0.526	0.77	0.474	0.476	0.967	0.42	0.084	**0.012**	0.473
Av. FC	-0.057	-0.031	0.048	-0.101	-0.002	-0.042	0.056	-0.096	0.039
N Genes	2	2	2	2	2	2	2	2	2

Signaling by TGF-beta Receptor Complex in Cancer (R-HSA-3304351)

	CORE	UCEC	BRCA	KIRC	KIRP	LUAD	LIHC	STAD	THCA
Regulation	Down	Down	Up	Down	Down	Down	Up	**Down**	Up
FDR	0.526	0.77	0.474	0.476	0.967	0.42	0.084	**0.012**	0.473
Av. FC	-0.057	-0.031	0.048	-0.101	-0.002	-0.042	0.056	-0.096	0.039
N Genes	2	2	2	2	2	2	2	2	2

SMAD4 MH2 Domain Mutants in Cancer (R-HSA-3311021)

	CORE	UCEC	BRCA	KIRC	KIRP	LUAD	LIHC	STAD	THCA
Regulation	Down	Down	Up	Down	Down	Down	Up	**Down**	Up
FDR	0.526	0.77	0.474	0.476	0.967	0.42	0.084	**0.012**	0.473
Av. FC	-0.057	-0.031	0.048	-0.101	-0.002	-0.042	0.056	-0.096	0.039
N Genes	2	2	2	2	2	2	2	2	2

SMAD2/3 MH2 Domain Mutants in Cancer (R-HSA-3315487)

	CORE	UCEC	BRCA	KIRC	KIRP	LUAD	LIHC	STAD	THCA
Regulation	Down	Down	Up	Down	Down	Down	Up	**Down**	Up
FDR	0.526	0.77	0.474	0.476	0.967	0.42	0.084	**0.012**	0.473
Av. FC	-0.057	-0.031	0.048	-0.101	-0.002	-0.042	0.056	-0.096	0.039
N Genes	2	2	2	2	2	2	2	2	2

FOXO-mediated transcription of cell cycle genes (R-HSA-9617828)

	CORE	UCEC	BRCA	KIRC	KIRP	LUAD	LIHC	STAD	THCA
Regulation	Down	Down	Up	Down	Up	Down	Up	**Down**	Up
FDR	0.516	0.529	0.397	0.269	0.75	0.637	0.094	**0.014**	0.704
Av. FC	-0.056	-0.045	0.02	-0.026	0.001	-0.046	0.029	-0.073	0.052
N Genes	6	6	6	6	6	6	6	6	6

SUMOylation of transcription cofactors (R-HSA-3899300)

	CORE	UCEC	BRCA	KIRC	KIRP	LUAD	LIHC	STAD	THCA
Regulation	Up	Up	Down	Down	Down	Down	Down	**Down**	Up
FDR	0.654	0.648	0.394	0.362	0.771	0.941	0.787	**0.024**	0.412
Av. FC	0.026	0.04	-0.1	-0.129	-0.009	-0.009	-0.045	-0.135	0.038
N Genes	1	1	1	1	1	1	1	1	1

Chaperone Mediated Autophagy (R-HSA-9613829)

	CORE	UCEC	BRCA	KIRC	KIRP	LUAD	LIHC	STAD	THCA
Regulation	Up	Up	Down	Down	Down	Down	Down	**Down**	Up
FDR	0.654	0.648	0.394	0.362	0.771	0.941	0.787	**0.024**	0.412
Av. FC	0.026	0.04	-0.1	-0.129	-0.009	-0.009	-0.045	-0.135	0.038
N Genes	1	1	1	1	1	1	1	1	1

Late endosomal microautophagy (R-HSA-9615710)

	CORE	UCEC	BRCA	KIRC	KIRP	LUAD	LIHC	STAD	THCA
Regulation	Up	Up	Down	Down	Down	Down	Down	**Down**	Up
FDR	0.654	0.648	0.394	0.362	0.771	0.941	0.787	**0.024**	0.412
Av. FC	0.026	0.04	-0.1	-0.129	-0.009	-0.009	-0.045	-0.135	0.038
N Genes	1	1	1	1	1	1	1	1	1

Aggrephagy (R-HSA-9646399)

	CORE	UCEC	BRCA	KIRC	KIRP	LUAD	LIHC	STAD	THCA
Regulation	Up	Up	Down	Down	Down	Down	Down	**Down**	Up
FDR	0.654	0.648	0.394	0.362	0.771	0.941	0.787	**0.024**	0.412
Av. FC	0.026	0.04	-0.1	-0.129	-0.009	-0.009	-0.045	-0.135	0.038
N Genes	1	1	1	1	1	1	1	1	1

Signaling by NODAL (R-HSA-1181150)

	CORE	UCEC	BRCA	KIRC	KIRP	LUAD	LIHC	STAD	THCA
Regulation	Down	Up	Up	Up	Down	Down	Up	**Down**	Up
FDR	0.588	0.585	0.568	0.054	0.986	0.469	0.054	**0.028**	0.766
Av. FC	-0.02	0.002	0.046	0.018	-0.003	-0.012	0.014	-0.052	0.026
N Genes	3	3	3	3	3	3	3	3	3

FOXO-mediated transcription of oxidative stress, metabolic and neuronal genes (R-HSA-9615017)

	CORE	UCEC	BRCA	KIRC	KIRP	LUAD	LIHC	STAD	THCA
Regulation	Down	Up	Up	Up	Down	Down	Up	**Down**	Up
FDR	0.588	0.585	0.568	0.054	0.986	0.469	0.054	**0.028**	0.766
Av. FC	-0.02	0.002	0.046	0.018	-0.003	-0.012	0.014	-0.052	0.026
N Genes	3	3	3	3	3	3	3	3	3

Downregulation of TGF-beta receptor signaling (R-HSA-2173788)

	CORE	UCEC	BRCA	KIRC	KIRP	LUAD	LIHC	STAD	THCA
Regulation	Down	Down	Up	Down	Down	Down	Up	**Down**	Up
FDR	0.318	0.404	0.215	0.296	0.826	0.298	0.067	**0.046**	0.347
Av. FC	-0.092	-0.066	0.121	-0.136	-0.001	-0.122	0.163	-0.121	0.055
N Genes	1	1	1	1	1	1	1	1	1

SMAD2/3 Phosphorylation Motif Mutants in Cancer (R-HSA-3304356)

	CORE	UCEC	BRCA	KIRC	KIRP	LUAD	LIHC	STAD	THCA
Regulation	Down	Down	Up	Down	Down	Down	Up	**Down**	Up
FDR	0.318	0.404	0.215	0.296	0.826	0.298	0.067	**0.046**	0.347
Av. FC	-0.092	-0.066	0.121	-0.136	-0.001	-0.122	0.163	-0.121	0.055
N Genes	1	1	1	1	1	1	1	1	1

Activation, translocation and oligomerization of BAX (R-HSA-114294)

	CORE	UCEC	BRCA	KIRC	KIRP	LUAD	LIHC	STAD	THCA
Regulation	Down	Up	Up	Up	Down	Down	Up	Up	**Up**
FDR	0.456	0.5	0.373	0.087	0.203	0.056	0.881	0.44	**0.001**
Av. FC	-0.041	0.047	0.085	0.025	-0.03	-0.015	0.05	0.01	0.142
N Genes	2	2	2	2	2	2	2	2	2

Activation, myristolyation of BID and translocation to mitochondria (R-HSA-75108)

	CORE	UCEC	BRCA	KIRC	KIRP	LUAD	LIHC	STAD	THCA
Regulation	Down	Up	Up	Up	Up	Up	Up	Up	**Up**
FDR	0.178	0.158	0.345	0.077	0.256	0.097	0.685	0.209	**0.003**
Av. FC	-0.089	0.089	0.091	0.255	0.03	0.116	0.056	0.052	0.132
N Genes	1	1	1	1	1	1	1	1	1

Activation and oligomerization of BAK protein (R-HSA-111452)

	CORE	UCEC	BRCA	KIRC	KIRP	LUAD	LIHC	STAD	THCA
Regulation	Up	Up	Up	Up	Up	Up	Down	Up	**Up**
FDR	0.177	0.432	0.332	0.187	0.133	0.128	0.082	0.341	**0.003**
Av. FC	0.017	0.03	0.11	0.057	0.058	0.136	-0.056	0.046	0.124
N Genes	2	2	2	2	2	2	2	2	2

Antimicrobial peptides (R-HSA-6803157)

	CORE	UCEC	BRCA	KIRC	KIRP	LUAD	LIHC	STAD	THCA
Regulation	Down	Up	Up	Up	Up	Down	Up	Up	**Up**
FDR	0.226	0.613	0.335	0.821	0.134	0.798	0.102	0.196	**0.02**
Av. FC	-0.35	0.098	0.287	0.029	0.14	-0.054	0.288	0.222	0.411
N Genes	1	1	1	1	1	1	1	1	1

Rab regulation of trafficking (R-HSA-9007101)

	CORE	UCEC	BRCA	KIRC	KIRP	LUAD	LIHC	STAD	THCA
Regulation	Down	Up	Up	Down	Down	Up	Up	Up	**Up**
FDR	0.214	0.779	0.725	0.528	0.843	0.368	0.221	0.264	**0.02**
Av. FC	-0.048	0.01	0.034	-0.083	-0.003	0.024	0.077	0.022	0.008
N Genes	5	5	5	5	5	5	5	5	5

YAP1- and WWTR1 (TAZ)-stimulated gene expression (R-HSA-2032785)

	CORE	UCEC	BRCA	KIRC	KIRP	LUAD	LIHC	STAD	THCA
Regulation	Down	Down	Up	Up	Up	Down	Up	Up	**Up**
FDR	0.656	0.932	0.169	0.632	0.525	0.212	0.965	0.77	**0.02**
Av. FC	-0.033	-0.004	0.156	0.044	0.015	-0.161	0.022	0.013	0.147
N Genes	1	1	1	1	1	1	1	1	1

RUNX3 regulates YAP1-mediated transcription (R-HSA-8951671)

	CORE	UCEC	BRCA	KIRC	KIRP	LUAD	LIHC	STAD	THCA
Regulation	Down	Down	Up	Up	Up	Down	Up	Up	**Up**
FDR	0.656	0.932	0.169	0.632	0.525	0.212	0.965	0.77	**0.02**
Av. FC	-0.033	-0.004	0.156	0.044	0.015	-0.161	0.022	0.013	0.147
N Genes	1	1	1	1	1	1	1	1	1

EGR2 and SOX10-mediated initiation of Schwann cell myelination (R-HSA-9619665)

	CORE	UCEC	BRCA	KIRC	KIRP	LUAD	LIHC	STAD	THCA
Regulation	Down	Down	Up	Up	Up	Down	Up	Up	**Up**
FDR	0.656	0.932	0.169	0.632	0.525	0.212	0.965	0.77	**0.02**
Av. FC	-0.033	-0.004	0.156	0.044	0.015	-0.161	0.022	0.013	0.147
N Genes	1	1	1	1	1	1	1	1	1

Post-translational protein phosphorylation (R-HSA-8957275)

	CORE	UCEC	BRCA	KIRC	KIRP	LUAD	LIHC	STAD	THCA
Regulation	Down	Up	Up	Down	Down	Up	Up	Down	**Up**
FDR	0.282	0.747	0.541	0.442	0.408	0.707	0.964	0.347	**0.023**
Av. FC	-0.184	0.015	0.106	-0.001	-0.031	0.079	0.084	-0.005	0.225
N Genes	2	2	2	2	2	2	2	2	2

RUNX1 regulates transcription of genes involved in differentiation of HSCs (R-HSA-8939236)

	CORE	UCEC	BRCA	KIRC	KIRP	LUAD	LIHC	STAD	THCA
Regulation	Up	Up	Down	Up	Up	Down	Up	Up	**Up**
FDR	0.234	0.596	0.052	0.514	0.638	0.538	0.89	0.411	**0.031**
Av. FC	0.085	0.058	-0.203	0.112	0.025	-0.072	0.037	0.037	0.042
N Genes	2	2	2	2	2	2	2	2	2

Myogenesis (R-HSA-525793)

	CORE	UCEC	BRCA	KIRC	KIRP	LUAD	LIHC	STAD	THCA
Regulation	Down	Down	Down	Down	Up	Down	Up	Up	**Up**
FDR	0.413	0.284	0.375	0.344	0.712	0.624	0.9	0.268	**0.031**
Av. FC	-0.138	-0.14	-0.011	-0.034	0.015	-0.003	0.11	0.161	0.061
N Genes	2	2	2	2	2	2	2	2	2

NTRK3 as a dependence receptor (R-HSA-9603505)

	CORE	UCEC	BRCA	KIRC	KIRP	LUAD	LIHC	STAD	THCA
Regulation	Up	Up	Up	Down	Down	Down	Up	Down	**Up**
FDR	0.783	0.938	0.404	0.263	0.237	0.193	0.934	0.649	**0.041**
Av. FC	0.007	0.004	0.078	-0.205	-0.089	-0.146	0.043	-0.032	0.152
N Genes	1	1	1	1	1	1	1	1	1

Intrinsic Pathway for Apoptosis (R-HSA-109606)

	CORE	UCEC	BRCA	KIRC	KIRP	LUAD	LIHC	STAD	THCA
Regulation	Down	Up	Up	Down	Down	Up	Up	Down	**Up**
FDR	0.901	0.977	0.914	0.282	0.332	0.096	0.252	0.863	**0.046**
Av. FC	-0.03	0.034	0.016	-0.048	-0.002	0.032	0.016	-0.026	0.044
N Genes	13	13	13	13	13	13	13	13	13

Platelet degranulation (R-HSA-114608)

	CORE	UCEC	BRCA	KIRC	KIRP	LUAD	LIHC	STAD	THCA
Regulation	Down	Up	Up	Down	Down	Up	Up	Down	**Up**
FDR	0.4	0.792	0.902	0.784	0.514	0.853	0.258	0.288	**0.049**
Av. FC	-0.142	0.029	0.025	-0.082	-0.05	0.063	0.132	-0.077	0.031
N Genes	3	3	3	3	3	3	3	3	3

Adherens junctions interactions (R-HSA-418990)

	CORE	UCEC	BRCA	KIRC	KIRP	LUAD	LIHC	STAD	THCA
Regulation	Down	Down	Up	Up	Up	Down	Up	Up	**Up**
FDR	0.637	0.4	0.448	0.265	0.487	0.654	0.998	0.459	**0.05**
Av. FC	-0.01	-0.146	0.026	0.141	0.073	0	0.063	0.118	0.011
N Genes	4	4	4	4	4	4	4	4	4

BH3-only proteins associate with and inactivate anti-apoptotic BCL-2 members (R-HSA-111453)

	CORE	UCEC	BRCA	KIRC	KIRP	LUAD	LIHC	STAD	THCA
Regulation	Down	Up	Up	Up	Up	Up	Up	Up	**Down**
FDR	0.656	0.31	0.783	0.308	0.278	0.274	0.591	0.683	**0.008**
Av. FC	-0.054	0.094	0.006	0.06	0.009	0.101	0.031	0.013	-0.003
N Genes	4	4	4	4	4	4	4	4	4

Cell-cell junction organization (R-HSA-421270)

	CORE	UCEC	BRCA	KIRC	KIRP	LUAD	LIHC	STAD	THCA
Regulation	Up	Down	Up	Up	Up	Down	Up	Up	**Down**
FDR	0.72	0.619	0.214	0.125	0.258	0.835	0.92	0.676	**0.013**
Av. FC	0.018	-0.119	0.143	0.23	0.095	-0.004	0.145	0.1	-0.115
N Genes	5	5	5	5	5	5	5	5	5

TBC/RABGAPs (R-HSA-8854214)

	CORE	UCEC	BRCA	KIRC	KIRP	LUAD	LIHC	STAD	THCA
Regulation	Down	Up	Up	Down	Up	Up	Up	Up	**Down**
FDR	0.112	0.455	0.484	0.582	0.807	0.44	0.155	0.346	**0.016**
Av. FC	-0.018	0.029	0.093	-0.099	0.009	0.045	0.132	0.066	-0.035
N Genes	3	3	3	3	3	3	3	3	3

Response to elevated platelet cytosolic Ca2+ (R-HSA-76005)

	CORE	UCEC	BRCA	KIRC	KIRP	LUAD	LIHC	STAD	THCA
Regulation	Down	Up	Up	Down	Down	Up	Up	Down	**Down**
FDR	0.472	0.925	0.981	0.909	0.595	0.942	0.204	0.441	**0.021**
Av. FC	-0.081	0.015	0.02	-0.069	-0.046	0.04	0.139	-0.054	-0.009
N Genes	4	4	4	4	4	4	4	4	4

Synthesis of PIPs at the early endosome membrane (R-HSA-1660516)

	CORE	UCEC	BRCA	KIRC	KIRP	LUAD	LIHC	STAD	THCA
Regulation	Down	Down	Down	Up	Down	Down	Down	Up	**Down**
FDR	0.578	0.441	0.087	0.397	0.853	0.135	0.096	0.63	**0.022**
Av. FC	-0.09	-0.14	-0.574	0.261	-0.001	-0.327	-0.27	0.044	-0.155
N Genes	1	1	1	1	1	1	1	1	1

Synthesis of IP2, IP, and Ins in the cytosol (R-HSA-1855183)

	CORE	UCEC	BRCA	KIRC	KIRP	LUAD	LIHC	STAD	THCA
Regulation	Down	Down	Down	Up	Down	Down	Down	Up	**Down**
FDR	0.578	0.441	0.087	0.397	0.853	0.135	0.096	0.63	**0.022**
Av. FC	-0.09	-0.14	-0.574	0.261	-0.001	-0.327	-0.27	0.044	-0.155
N Genes	1	1	1	1	1	1	1	1	1

Transcriptional regulation by RUNX1 (R-HSA-8878171)

	CORE	UCEC	BRCA	KIRC	KIRP	LUAD	LIHC	STAD	THCA
Regulation	Up	Up	Down	Up	Up	Up	Down	Up	**Down**
FDR	0.216	0.538	0.479	0.623	0.668	0.668	0.955	0.504	**0.024**
Av. FC	0.065	0.007	-0.09	0.088	0.013	0.008	-0.03	0.041	-0.006
N Genes	6	6	6	6	6	6	6	6	6

BMAL1:CLOCK,NPAS2 activates circadian gene expression (R-HSA-1368108)

	CORE	UCEC	BRCA	KIRC	KIRP	LUAD	LIHC	STAD	THCA
Regulation	Down	Up	Up	Up	Down	Up	Up	Down	**Down**
FDR	0.606	0.561	0.836	0.817	0.356	0.571	0.397	0.692	**0.03**
Av. FC	-0.124	0.093	0.056	0.048	-0.078	0.19	0.313	-0.054	-0.237
N Genes	1	1	1	1	1	1	1	1	1

Circadian Clock (R-HSA-400253)

	CORE	UCEC	BRCA	KIRC	KIRP	LUAD	LIHC	STAD	THCA
Regulation	Down	Up	Up	Up	Down	Up	Up	Down	**Down**
FDR	0.606	0.561	0.836	0.817	0.356	0.571	0.397	0.692	**0.03**
Av. FC	-0.124	0.093	0.056	0.048	-0.078	0.19	0.313	-0.054	-0.237
N Genes	1	1	1	1	1	1	1	1	1

Dissolution of Fibrin Clot (R-HSA-75205)

	CORE	UCEC	BRCA	KIRC	KIRP	LUAD	LIHC	STAD	THCA
Regulation	Down	Up	Up	Up	Down	Up	Up	Down	**Down**
FDR	0.606	0.561	0.836	0.817	0.356	0.571	0.397	0.692	**0.03**
Av. FC	-0.124	0.093	0.056	0.048	-0.078	0.19	0.313	-0.054	-0.237
N Genes	1	1	1	1	1	1	1	1	1

Cell junction organization (R-HSA-446728)

	CORE	UCEC	BRCA	KIRC	KIRP	LUAD	LIHC	STAD	THCA
Regulation	Up	Down	Up	Up	Up	Down	Up	Up	**Down**
FDR	0.822	0.702	0.402	0.188	0.255	0.7	0.899	0.49	**0.045**
Av. FC	0.024	-0.11	0.12	0.171	0.069	-0.041	0.146	0.117	-0.091
N Genes	6	6	6	6	6	6	6	6	6

RMTs methylate histone arginines (R-HSA-3214858)

	CORE	UCEC	BRCA	KIRC	KIRP	LUAD	LIHC	STAD	THCA
Regulation	Down	Up	Down	Up	Down	Down	Up	Up	**Down**
FDR	0.731	0.234	0.938	0.471	0.577	0.854	0.965	0.644	**0.047**
Av. FC	-0.007	0.085	-0.011	0.034	-0.002	-0.016	0.01	0.007	-0.024
N Genes	2	2	2	2	2	2	2	2	2

Chromatin modifying enzymes (R-HSA-3247509)

	CORE	UCEC	BRCA	KIRC	KIRP	LUAD	LIHC	STAD	THCA
Regulation	Down	Up	Down	Up	Down	Down	Up	Up	**Down**
FDR	0.731	0.234	0.938	0.471	0.577	0.854	0.965	0.644	**0.047**
Av. FC	-0.007	0.085	-0.011	0.034	-0.002	-0.016	0.01	0.007	-0.024
N Genes	2	2	2	2	2	2	2	2	2

Chromatin organization (R-HSA-4839726)

	CORE	UCEC	BRCA	KIRC	KIRP	LUAD	LIHC	STAD	THCA
Regulation	Down	Up	Down	Up	Down	Down	Up	Up	**Down**
FDR	0.731	0.234	0.938	0.471	0.577	0.854	0.965	0.644	**0.047**
Av. FC	-0.007	0.085	-0.011	0.034	-0.002	-0.016	0.01	0.007	-0.024
N Genes	2	2	2	2	2	2	2	2	2

Differently regulated pathways

SUMOylation (R-HSA-2990846)

	CORE	UCEC	BRCA	KIRC	KIRP	LUAD	LIHC	STAD	THCA
Regulation	**Down**	Down	Down	**Down**	Down	Up	Down	**Up**	Down
FDR	**$<$ 0.001**	0.967	0.408	**0.013**	0.474	0.544	0.372	**0.037**	0.815
Av. FC	-0.061	-0.027	-0.155	-0.01	-0.004	0.08	-0.046	0.01	-0.014
N Genes	8	8	8	8	8	8	8	8	8

SUMO E3 ligases SUMOylate target proteins (R-HSA-3108232)

	CORE	UCEC	BRCA	KIRC	KIRP	LUAD	LIHC	STAD	THCA
Regulation	**Down**	Down	Down	**Down**	Down	Up	Down	**Up**	Down
FDR	**$<$ 0.001**	0.967	0.408	**0.013**	0.474	0.544	0.372	**0.037**	0.815
Av. FC	-0.061	-0.027	-0.155	-0.01	-0.004	0.08	-0.046	0.01	-0.014
N Genes	8	8	8	8	8	8	8	8	8

HSP90 chaperone cycle for steroid hormone receptors (SHR) (R-HSA-3371497)

	CORE	UCEC	BRCA	KIRC	KIRP	LUAD	LIHC	STAD	THCA
Regulation	**Down**	Up	Down	**Up**	Up	Up	Down	**Up**	Down
FDR	**< 0.001**	0.984	0.097	**0.001**	0.381	0.303	0.279	**0.011**	0.968
Av. FC	-0.063	0.004	-0.242	0.022	0.067	0.02	-0.112	0.062	-0.003
N Genes	2	2	2	2	2	2	2	2	2

Activated PKN1 stimulates transcription of AR (androgen receptor) regulated genes KLK2 and KLK3 (R-HSA-5625886)

	CORE	UCEC	BRCA	KIRC	KIRP	LUAD	LIHC	STAD	THCA
Regulation	**Down**	Up	Down	Down	Up	Down	Down	**Up**	Up
FDR	**0.004**	0.92	0.201	0.161	0.2	0.739	0.6	**0.013**	0.879
Av. FC	-0.295	0.015	-0.342	-0.316	0.121	-0.053	-0.139	0.182	0.005
N Genes	1	1	1	1	1	1	1	1	1

Homology Directed Repair (R-HSA-5693538)

	CORE	UCEC	BRCA	KIRC	KIRP	LUAD	LIHC	STAD	THCA
Regulation	**Down**	**Up**	**Up**	**Up**	Up	**Up**	Up	Up	Up
FDR	**0.027**	**0.015**	**0.034**	**0.011**	0.424	**0.01**	0.731	0.203	0.163
Av. FC	-0.021	0.014	0.042	0.03	0.009	0.071	0.02	0.044	0.038
N Genes	10	10	10	10	10	10	10	10	10

Cellular Senescence (R-HSA-2559583)

	CORE	UCEC	BRCA	KIRC	KIRP	LUAD	LIHC	STAD	THCA
Regulation	Down	**Down**	Up	Down	Down	**Up**	Up	Up	Up
FDR	0.196	**0.035**	0.197	0.186	0.075	**0.038**	0.257	0.765	0.604
Av. FC	-0.071	-0.008	0.042	-0.055	-0.025	0.053	0.039	0.034	0.042
N Genes	11	11	11	11	11	11	11	11	11

The role of GTSE1 in G2/M progression after G2 checkpoint (R-HSA-8852276)

	CORE	UCEC	BRCA	KIRC	KIRP	LUAD	LIHC	STAD	THCA
Regulation	Down	Up	**Up**	Up	Down	Up	**Down**	Down	Up
FDR	0.256	0.21	**0.028**	0.243	0.859	0.199	**0.029**	0.602	0.838
Av. FC	-0.051	0.112	0.091	0.213	-0.001	0.007	-0.041	-0.013	0.027
N Genes	4	4	4	4	4	4	4	4	4

Integrin cell surface interactions (R-HSA-216083)

	CORE	UCEC	BRCA	KIRC	KIRP	LUAD	LIHC	STAD	THCA
Regulation	Up	Down	Down	Up	**Up**	Down	Up	**Down**	Up
FDR	0.623	0.895	0.81	0.581	**0.034**	0.956	0.595	**0.036**	0.469
Av. FC	0.007	-0.071	-0.052	0.094	0.053	-0.028	0.049	-0.063	0.036
N Genes	5	5	5	5	5	5	5	5	5

Mitotic G2-G2/M phases (R-HSA-453274)

	CORE	UCEC	BRCA	KIRC	KIRP	LUAD	LIHC	STAD	THCA
Regulation	Down	Up	Up	**Up**	Down	Up	**Down**	Down	Up
FDR	0.268	0.173	0.325	**0.023**	0.925	0.09	**0.05**	0.448	0.785
Av. FC	-0.001	0.119	0.057	0.242	-0.002	0.046	-0.061	-0.012	0.036
N Genes	6	6	6	6	6	6	6	6	6

G2/M Transition (R-HSA-69275)

	CORE	UCEC	BRCA	KIRC	KIRP	LUAD	LIHC	STAD	THCA
Regulation	Down	Up	Up	**Up**	Down	Up	**Down**	Down	Up
FDR	0.268	0.173	0.325	**0.023**	0.925	0.09	**0.05**	0.448	0.785
Av. FC	-0.001	0.119	0.057	0.242	-0.002	0.046	-0.061	-0.012	0.036
N Genes	6	6	6	6	6	6	6	6	6

Inhibition of TSC complex formation by PKB (R-HSA-165181)

	CORE	UCEC	BRCA	KIRC	KIRP	LUAD	LIHC	STAD	THCA
Regulation	Up	Up	Up	Up	Up	Up	**Up**	Up	**Down**
FDR	0.727	0.504	0.19	0.812	0.753	0.466	**0.029**	0.337	**0.026**
Av. FC	0.08	0.076	0.132	0.021	0.006	0.104	0.187	0.077	-0.109
N Genes	2	2	2	2	2	2	2	2	2

Protein / Gene level results

Classical differential expression analysis performed on the genes / proteins of every dataset.

Note: Depending on the gene set analysis method used, the approach used to assess differential expression at the gene / protein level may vary to the approach used for the pathway level.

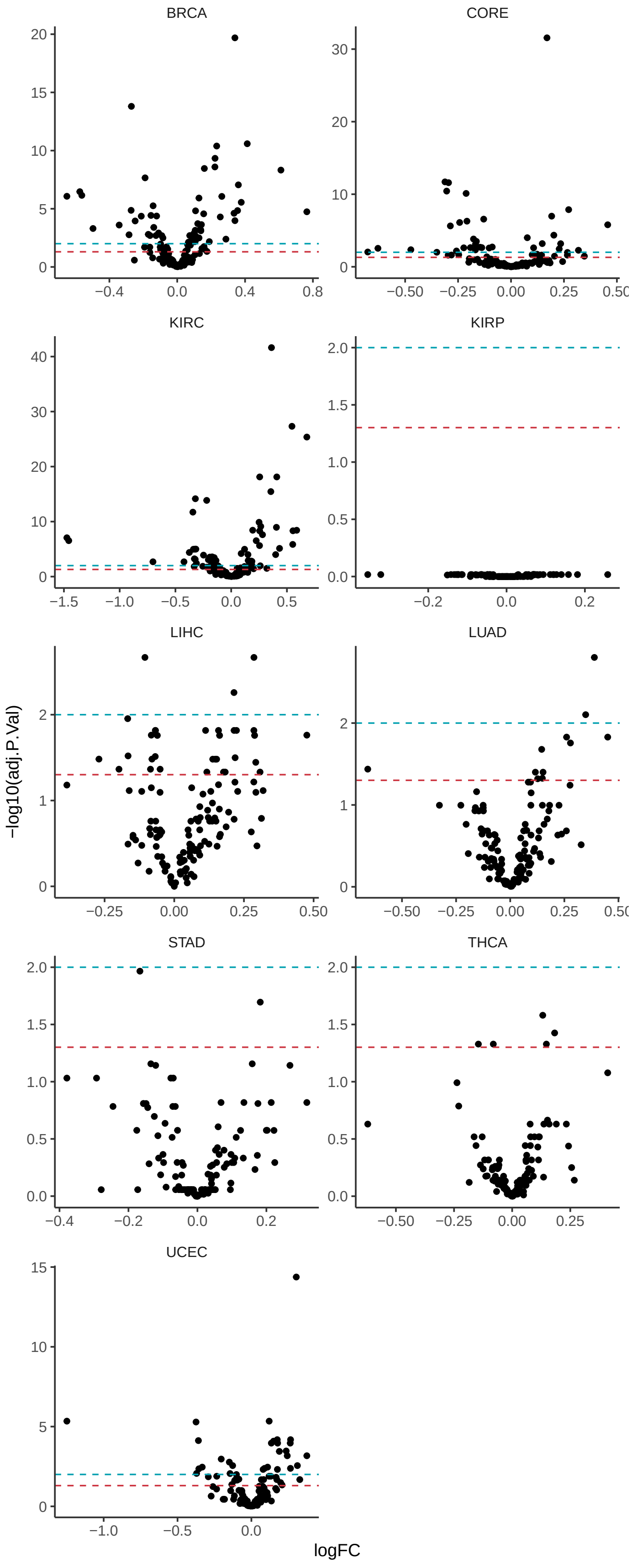

BRCA
CORE
KIRC
KIRP
LIHC
LUAD
STAD
THCA
UCEC
−log10(adj.P.Val)
logFC

Top up-regulated protein/genes

CORE

Identifier	logFC	AveExpr	t	P.Value	adj.P.Val	B
P06401	0.1698598	-0.9045014	13.226236	0.0000000	0.0000000	67.3254462
P03372	0.2721933	-2.2230346	6.311621	0.0000000	0.0000000	11.7913588
P51587	0.1919369	-0.3817945	5.931061	0.0000000	0.0000001	9.6320381
Q13085	0.4566365	1.5020192	5.347163	0.0000001	0.0000016	6.5471327
P23771	0.2023138	-0.2690842	4.643707	0.0000044	0.0000439	3.2113859
Q9UJM3	0.0768768	-0.2805956	4.450699	0.0000106	0.0000983	2.3711443
Q13490	0.1475038	0.3028166	3.967728	0.0000833	0.0006372	0.4137121
P43246	0.2344682	-0.9291391	3.946563	0.0000908	0.0006557	0.3327339
P16949	0.1066893	-0.3709595	3.526753	0.0004599	0.0024363	-1.1889583
Q08050	0.2276867	-0.6678785	3.396594	0.0007374	0.0034236	-1.6277788

UCEC

Identifier	logFC	AveExpr	t	P.Value	adj.P.Val	B
Q06609	0.3039634	-1.0306181	8.795795	0.00e+00	0.0000000	28.427658
P49959	0.1204203	-0.0636266	5.407341	1.00e-07	0.0000046	7.134785
O14497	0.1752180	-0.1207953	4.752964	2.70e-06	0.0000660	4.020793
Q08050	0.2652416	-0.7180668	4.728964	3.00e-06	0.0000660	3.913411
P16949	0.1502452	-0.2534793	4.620569	5.00e-06	0.0000818	3.434547
Q13541	0.2631746	0.1888165	4.531194	7.60e-06	0.0001087	3.047284
P51587	0.1351715	-0.2279471	4.491020	9.10e-06	0.0001087	2.875447
P42336	0.1776240	-0.6017652	4.487839	9.20e-06	0.0001087	2.861906
O43521	0.2356193	0.2245165	4.207823	3.13e-05	0.0003387	1.703934
P35568	0.1892512	-0.6198825	4.175528	3.59e-05	0.0003586	1.574795

BRCA

Identifier	logFC	AveExpr	t	P.Value	adj.P.Val	B
Q06609	0.3401400	-1.1541719	10.037809	0e+00	0.0e+00	40.351017
P08243	0.4129098	-0.8687461	7.310555	0e+00	0.0e+00	18.735354
P16949	0.2327343	-0.1754943	7.203762	0e+00	0.0e+00	18.008790

Q9NQ88	0.2229877	0.6850063	6.811684	0e+00	0.0e+00	15.423196
Q92574	0.2222093	1.5540602	6.520394	0e+00	0.0e+00	13.586641
P06493	0.1599406	0.1137014	6.448176	0e+00	0.0e+00	13.142577
O95471	0.6114499	1.1173322	6.376044	0e+00	0.0e+00	12.703530
Q13315	0.3605166	2.0383558	5.852748	0e+00	1.0e-07	9.654121
P06239	0.2629636	0.3161711	5.383622	1e-07	9.0e-07	7.126153
Q15831	0.1282455	0.4412769	5.310263	1e-07	1.2e-06	6.748679

KIRC

Identifier	logFC	AveExpr	t	P.Value	adj.P.Val	B
P06401	0.3614138	-1.0932374	15.559926	0	0	90.29254
Q08050	0.5450567	-1.1385445	12.168019	0	0	56.91718
Q06609	0.6785585	-0.9910971	11.636749	0	0	52.04912
P35568	0.4090119	-0.7848760	9.659973	0	0	35.08597
O43524	0.2557767	-0.6552265	9.652802	0	0	35.02812
P16949	0.3557190	-0.2113600	8.855572	0	0	28.78256
Q15831	0.2500415	0.4495822	6.975048	0	0	15.65945
P43246	0.2653851	-1.2470281	6.688848	0	0	13.88051
P04637	0.4057569	-1.6577482	6.611965	0	0	13.41313
P49959	0.1930475	0.1044157	6.399771	0	0	12.14659

KIRP

Identifier	logFC	AveExpr	t	P.Value	adj.P.Val	B
P17301	0.1188196	-0.3101658	2.550991	0.0114375	0.9610855	-2.812658
P46527	0.0936450	0.3964034	2.066148	0.0400142	0.9610855	-3.743169
P12830	0.2582271	-0.4397216	1.765422	0.0789138	0.9610855	-4.226402
P06730	0.0777850	0.0783874	1.687100	0.0930359	0.9610855	-4.340234
P16949	0.0558864	-0.1072599	1.584556	0.1145385	0.9610855	-4.481686
P57735	0.1580401	-2.2403613	1.552404	0.1220378	0.9610855	-4.524260
O95471	0.1810929	0.8714475	1.534228	0.1264455	0.9610855	-4.547953
Q02750	0.1274700	-0.1457065	1.415602	0.1583406	0.9610855	-4.695884
P10721	0.0679527	-0.1377624	1.412975	0.1591105	0.9610855	-4.699027

P46531	0.0498844	-0.6034691	1.355374	0.1767222	0.9610855	-4.766534

LUAD

Identifier	logFC	AveExpr	t	P.Value	adj.P.Val	B
Q06609	0.3887259	-0.8625434	4.436190	0.0000121	0.0015778	3.0075824
Q13315	0.3484721	1.8129984	3.885062	0.0001215	0.0078948	0.9015692
P08243	0.4503135	-0.5004417	3.555809	0.0004264	0.0148428	-0.2343436
P17861	0.2604050	-1.0343843	3.537089	0.0004567	0.0148428	-0.2961259
Q8TCU6	0.2781835	-0.9999826	3.429703	0.0006733	0.0175056	-0.6446522
P46531	0.1452779	-0.7779665	3.327135	0.0009666	0.0209433	-0.9681557
P42336	0.1515964	-0.7494578	3.014435	0.0027545	0.0397961	-1.8972393
P55957	0.1162268	0.5073136	3.014368	0.0027551	0.0397961	-1.8974274
P35568	0.1484050	-0.4674346	2.927042	0.0036363	0.0472725	-2.1413622
P16949	0.1268253	-0.2707854	2.892357	0.0040527	0.0478960	-2.2363535

LIHC

Identifier	logFC	AveExpr	t	P.Value	adj.P.Val	B
P52701	0.2859627	0.0864017	4.256952	0.0000331	0.0021516	2.1242982
P13010	0.2142906	1.4327893	3.915216	0.0001274	0.0055222	0.8968914
Q96T37	0.2857233	1.8691785	3.438598	0.0007244	0.0152589	-0.6694218
P28482	0.2232452	0.9476618	3.282710	0.0012320	0.0152589	-1.1433873
P04049	0.1123868	1.1742102	3.280792	0.0012400	0.0152589	-1.1490981
P49815	0.2147320	1.3914999	3.278262	0.0012505	0.0152589	-1.1566275
Q92574	0.1587630	1.8114303	3.268692	0.0012911	0.0152589	-1.1850568
O95471	0.4757491	1.2789485	3.179937	0.0017308	0.0173084	-1.4452263
P84022	0.1626794	0.7565267	3.139304	0.0019753	0.0174437	-1.5622075
HSP70	0.2883547	0.6609645	3.114530	0.0021397	0.0174437	-1.6328719

STAD

Identifier	logFC	AveExpr	t	P.Value	adj.P.Val	B
P10275	0.1822273	-0.7991901	3.638846	0.0003106	0.0201914	0.1015787
O14757	0.1588903	-0.2649299	3.162479	0.0016865	0.0697939	-1.3951940

O00418	0.2683399	-0.4496252	3.001210	0.0028617	0.0720436	-1.8569544
P04626	0.3173069	0.4306360	2.496116	0.0129675	0.1520487	-3.1532555
Q13315	0.2140206	1.7519806	2.465780	0.0141003	0.1520487	-3.2238019
P12931	0.1348246	-0.2828929	2.430016	0.0155478	0.1520487	-3.3058956
P04049	0.0684578	0.8510417	2.410891	0.0163745	0.1520487	-3.3493217
Q9UQC2	0.1755257	0.5837496	2.308548	0.0214900	0.1552057	-3.5760355
P23771	0.0601558	-0.3136340	1.986584	0.0476667	0.2478669	-4.2267524
P60484	0.1998798	0.1813740	1.935455	0.0536558	0.2661734	-4.3213112

THCA

Identifier	logFC	AveExpr	t	P.Value	adj.P.Val	B
P55957	0.1316510	0.6743958	3.753052	0.0002021	0.0262771	0.5058534
P19022	0.1827264	0.2942489	3.471591	0.0005772	0.0375157	-0.4199964
P46937	0.1472141	0.6494144	3.238331	0.0013086	0.0469273	-1.1361161
HSP70	0.4109777	1.1775538	2.907438	0.0038589	0.0836087	-2.0711446
Q07812	0.1519597	1.1928212	2.443774	0.0149909	0.2165345	-3.2190464
P49959	0.0773897	0.2468752	2.320181	0.0208627	0.2345362	-3.4926027
Q15121	0.1363817	0.7406182	2.295832	0.0222320	0.2345362	-3.5448741
P13639	0.1592940	0.6703278	2.289623	0.0225935	0.2345362	-3.5581170
Q92878	0.1899728	0.3264650	2.232055	0.0261965	0.2345362	-3.6792448
Q5SQI0	0.2330742	1.5338777	2.219261	0.0270619	0.2345362	-3.7057570

Top down-regulated protein/genes

CORE

Identifier	logFC	AveExpr	t	P.Value	adj.P.Val	B
P07992	-0.3118009	-0.2686805	-7.828354	0.00e+00	0.0000000	21.492761
P10275	-0.2950646	-0.7992665	-7.734103	0.00e+00	0.0000000	20.841362
P38936	-0.3038855	0.4357178	-7.302622	0.00e+00	0.0000000	17.939047
P62491	-0.2119545	-0.2892597	-7.157983	0.00e+00	0.0000000	16.996042
P11836	-0.1290454	-0.2129519	-5.738364	0.00e+00	0.0000003	8.583011
Q06830	-0.2081443	-1.6611031	-5.599327	0.00e+00	0.0000005	7.844935
O95661	-0.2425107	-2.6492359	-5.502762	1.00e-07	0.0000008	7.341702

O14757	-0.2864426	-0.0867553	-5.259158	2.00e-07	0.0000023	6.106721
P11413	-0.1770870	-1.1222228	-4.342444	1.71e-05	0.0001483	1.914265
P18887	-0.1635948	-0.0151304	-4.151008	3.90e-05	0.0003168	1.131900

UCEC

Identifier	logFC	AveExpr	t	P.Value	adj.P.Val	B
P35749	-1.2490684	0.4590102	-5.408379	0.0000001	0.0000046	7.1400045
P14921	-0.3746596	0.4187933	-5.328200	0.0000002	0.0000052	6.7393470
P13639	-0.3586228	1.3403325	-4.666950	0.0000041	0.0000755	3.6382178
Q92878	-0.2041803	0.4526459	-3.854085	0.0001335	0.0010844	0.3397739
Q13131	-0.1492049	0.9676027	-3.724897	0.0002208	0.0016886	-0.1305796
P38936	-0.1267729	0.2915281	-3.563966	0.0004053	0.0027901	-0.6954258
Q53EL6	-0.3327534	1.5477547	-3.479429	0.0005526	0.0034726	-0.9827143
P35222	-0.3554753	2.6225046	-3.370664	0.0008160	0.0044201	-1.3427310
P04406	-0.3709842	2.2189665	-3.143481	0.0017825	0.0085823	-2.0596214
P35240	-0.1442540	1.2476682	-3.125833	0.0018905	0.0087772	-2.1133156

BRCA

Identifier	logFC	AveExpr	t	P.Value	adj.P.Val	B
O95661	-0.2701732	-2.7791924	-8.358942	0.0e+00	0.00e+00	26.362381
P38936	-0.1894245	0.4782336	-6.106572	0.0e+00	0.00e+00	11.103291
O15327	-0.5743739	0.1733981	-5.599471	0.0e+00	3.00e-07	8.264884
P23771	-0.5625465	1.8442552	-5.450900	1.0e-07	7.00e-07	7.476601
P49327	-0.6506406	1.4214765	-5.402104	1.0e-07	8.00e-07	7.222018
P06401	-0.1421236	-0.5522058	-4.990110	7.0e-07	5.60e-06	5.158346
P57735	-0.2718180	-1.3473176	-4.796996	1.9e-06	1.36e-05	4.244256
P07992	-0.1547152	-0.4777139	-4.523710	6.9e-06	3.74e-05	3.009288
O14757	-0.1214914	-0.4066509	-4.489107	8.1e-06	4.21e-05	2.857844
P35968	-0.2119256	1.8898465	-4.474142	8.7e-06	4.33e-05	2.792693

KIRC

Identifier	logFC	AveExpr	t	P.Value	adj.P.Val	B

P11836	-0.3211419	0.3384579	-8.441881	0.00e+00	0.0000000	25.693616
P07992	-0.2195563	-0.2519220	-8.330243	0.00e+00	0.0000000	24.878691
Q92878	-0.3435219	0.2200747	-7.624971	0.00e+00	0.0000000	19.921452
P04406	-1.4735341	3.3317258	-5.799460	0.00e+00	0.0000001	8.754191
P35749	-1.4567874	1.7047932	-5.569538	0.00e+00	0.0000003	7.531504
P10275	-0.3164990	0.0254505	-4.829143	1.80e-06	0.0000100	3.893309
Q13131	-0.3235710	1.6722147	-4.819883	1.90e-06	0.0000100	3.850758
P62491	-0.3386858	0.0839660	-4.790467	2.20e-06	0.0000107	3.716083
P43405	-0.3753021	1.6587633	-4.492172	8.90e-06	0.0000411	2.392909
P14921	-0.2480704	0.8239695	-4.207667	3.08e-05	0.0001252	1.203884

KIRP

Identifier	logFC	AveExpr	t	P.Value	adj.P.Val	B
Q5SQI0	-0.3543983	2.5711226	-2.340014	0.0202005	0.9610855	-3.240229
P24864	-0.1132769	-0.9138394	-2.096017	0.0372518	0.9610855	-3.691207
P43246	-0.1292972	-0.5680950	-2.037364	0.0428400	0.9610855	-3.792571
Q15118	-0.0786337	-0.2111651	-1.928440	0.0551188	0.9610855	-3.973500
P13639	-0.1233753	0.6140301	-1.867909	0.0631367	0.9610855	-4.069917
P49327	-0.1419088	-0.5215990	-1.752617	0.0810947	0.9610855	-4.245356
P18065	-0.1261368	-0.3456619	-1.738525	0.0835513	0.9610855	-4.266057
Q9UJM3	-0.0642351	0.1856125	-1.656370	0.0991071	0.9610855	-4.383527
Q13085	-0.1336519	1.4166211	-1.569489	0.1180061	0.9610855	-4.501743
P28482	-0.1149914	0.8579071	-1.496475	0.1359981	0.9610855	-4.596293

LUAD

Identifier	logFC	AveExpr	t	P.Value	adj.P.Val	B
P35749	-0.6581334	0.8435567	-3.117743	0.0019670	0.0365299	-1.599874
P38936	-0.1553590	0.2310125	-2.668835	0.0079511	0.0689093	-2.822539
O15327	-0.3271059	-0.5551664	-2.462620	0.0142536	0.1008876	-3.323155
P45984	-0.1249517	0.4628919	-2.401255	0.0168383	0.1008876	-3.464625
P49023	-0.2280996	0.5526838	-2.379497	0.0178493	0.1008876	-3.513957
P46937	-0.1609803	0.4386852	-2.338749	0.0198873	0.1077227	-3.605174

Q13131	-0.1245541	0.9946575	-2.320921	0.0208413	0.1083747	-3.644605
Q15797	-0.1631389	0.1638621	-2.269926	0.0237944	0.1178209	-3.755769
Q07812	-0.1461831	1.3169636	-2.259034	0.0244705	0.1178209	-3.779203
Q92997	-0.1238775	0.4961552	-2.228641	0.0264460	0.1183164	-3.844012

LIHC

Identifier	logFC	AveExpr	t	P.Value	adj.P.Val	B
P37023	-0.1052297	-0.3888135	-4.277449	0.0000305	0.0021516	2.2005832
Q16611	-0.1670349	1.1856389	-3.650035	0.0003422	0.0111207	0.0039537
P01111	-0.0673326	-1.3927496	-3.411558	0.0007953	0.0152589	-0.7530141
P06493	-0.0679584	0.0970100	-3.302812	0.0011517	0.0152589	-1.0833551
Q07955	-0.0823727	-1.5687027	-3.185965	0.0016970	0.0173084	-1.4277567
P31946	-0.0607372	-0.4814305	-3.113484	0.0021469	0.0174437	-1.6358420
O95661	-0.1657231	-2.7311466	-2.919187	0.0039513	0.0302156	-2.1723980
O43524	-0.0682077	-0.5032268	-2.895006	0.0042540	0.0307236	-2.2369861
O15327	-0.2702279	-0.7578147	-2.835170	0.0050966	0.0329202	-2.3947102
P16284	-0.0799460	-1.1569044	-2.820958	0.0053179	0.0329202	-2.4317316

STAD

Identifier	logFC	AveExpr	t	P.Value	adj.P.Val	B
Q06830	-0.1667870	-1.6186272	-3.976683	0.0000833	0.0108251	1.281196
Q99497	-0.1350570	0.6362687	-3.089624	0.0021475	0.0697939	-1.606640
P84022	-0.1211047	0.7030917	-2.954145	0.0033251	0.0720436	-1.987380
Q9UJM3	-0.0770557	-0.1278675	-2.765574	0.0059519	0.0930445	-2.490162
P04406	-0.2926288	1.0143459	-2.751037	0.0062172	0.0930445	-2.527601
P16284	-0.0700309	-1.2180812	-2.732534	0.0065703	0.0930445	-2.574983
Q03135	-0.3786200	2.2587708	-2.703676	0.0071573	0.0930445	-2.648265
P35968	-0.1488797	1.4106419	-2.354287	0.0190526	0.1552057	-3.475893
Q13541	-0.1566766	0.3920106	-2.344240	0.0195661	0.1552057	-3.498054
P17301	-0.1562394	-0.2810261	-2.317032	0.0210183	0.1552057	-3.557604

THCA

Identifier	logFC	AveExpr	t	P.Value	adj.P.Val	B
P49815	-0.1452619	0.9964486	-3.182742	0.0015796	0.0469273	-1.299860
O14497	-0.0810571	0.1327141	-3.142889	0.0018049	0.0469273	-1.415600
P05121	-0.2373063	-2.6083141	-2.792735	0.0054921	0.1019959	-2.372877
Q6R327	-0.2297824	0.0267141	-2.587423	0.0100420	0.1631831	-2.883871
O95471	-0.6211137	2.4605788	-2.263964	0.0241423	0.2345362	-3.612477
P17252	-0.1288605	-0.5284884	-2.043312	0.0417143	0.3029403	-4.055297
P42229	-0.1636961	1.4762001	-1.975515	0.0489365	0.3029403	-4.182461
O15327	-0.1554454	-0.8929613	-1.838750	0.0667360	0.3614869	-4.426195
P15056	-0.1021615	1.0343206	-1.612373	0.1077151	0.4822770	-4.791882
P10415	-0.1185199	0.1834665	-1.580947	0.1147263	0.4822770	-4.838913
